# MCMC-Driven mathematical modeling of the impact of HPV vaccine uptake in reducing cervical cancer

**DOI:** 10.1016/j.sciaf.2025.e02633

**Published:** 2025-06

**Authors:** Sylas Oswald, Eunice Mureithi, Berge Tsanou, Michael Chapwanya, Kijakazi Mashoto, Crispin Kahesa

**Affiliations:** a Department of Mathematics, University of Dar-es-Salaam, P.O Box 35062, Dar-es-salaam, Tanzania; b Department of Mathematics & Computer Science, University of Dschang, Cameroon; c Department of Mathematics & Applied Mathematics, University of Pretoria, South Africa; d National Institute for Medical Research, Headquarters: Dar Es Salaam (NIMR), Tanzania

**Keywords:** Trivial node, Digraph, Self-loop, CIN1,2,3, Markov chain, Parameter identifiability, MCMC ran, Mean posterior, Herd immunity

## Abstract

Human Papillomavirus (HPV) is a group of contagious viruses primarily transmitted through sexual contact and is a major cause of severe health issues, including cervical cancer. In Sub-Saharan Africa, including Tanzania, cervical cancer is the leading cause of cancer-related deaths among women of all ages. In 2022, there were 125,699 new cases and 80,614 deaths, making cervical cancer the second most common cancer. Of these, Tanzania recorded 10,868 cases and 6,832 deaths. To reduce the number of girls and female affected by HPV infections, particularly those vulnerable to cervical cancer, we have developed and analyzed a mathematical model for HPV transmission dynamics that incorporates vaccination. The analysis demonstrates the presence of both HPV-free and endemic equilibrium states. By applying the Graph Theoretic method, the reproduction number $R_{e}$ was computed. The results indicate that the HPV-free equilibrium is globally asymptotically stable when $R_{e}\leq 1$, while the endemic equilibrium is globally asymptotically stable when $R_{e}>1$. We employed a Markov Chain Monte Carlo (MCMC) method for model calibration, which highlighted several key factors. The interaction between vaccination rates for young girls and older females suggests long-term benefits from vaccinating both groups, contributing to increased herd immunity. Additionally, the strong identifiability of the recovery rate emphasizes its critical role in reducing HPV prevalence and cervical cancer progression. The correlations observed indicate the dual role of vaccination in both preventing infection and promoting recovery. On the other hand, the poor identifiability of the mortality rate points to gaps in understanding the long-term burden of cervical cancer. However, since the data used are synthetic, the uncertainties highlight how important it is to use real data and break it into groups to better understand how different factors affect the results. The herd immunity threshold was calculated to be 0.4417, recommending that at least 55.83% of the population be vaccinated to halt HPV transmission and reduce cervical cancer incidence.

## Introduction

Human Papillomavirus (HPV) is a group of contagious and life-threatening viruses that spread from person to person and often cause significant health problems, one of the most severe being cervical cancer [1], [2].

HPV is a small double-stranded DNA virus, with genome lengths ranging from 5748 bp for Sparus aurata papillomavirus type 1 (SaPV1) to 8607 bp for canine papillomavirus type 1 (CPV1). There are over 400 subtypes, more than 40 of which infects the genital and anal areas in humans, and these subtypes are classified into five genera α, β, γ, ν, and μ papillomaviruses, which account for 65, 54, 99, 1, and 3 HPV types, respectively [3], [4], [5], [6]. Infection with subtypes of HPV causes pathological changes, including cancer. Human papillomavirus (HPV) is mainly transmitted through direct skin-to-skin contact, most often during sexual activity. Nearly all sexually active individuals will be exposed to the virus at some point in their lives. While HPV infections usually clear from the body on their own, some cases persist. Persistent infection with high-risk HPV strains is a significant factor in the development of cervical cancer. The progression to cervical cancer is complex and stepwise process involving increasingly severe precancerous changes, known as Cervical Intraepithelial Neoplasia (CIN), which is traditionally divided into three histopathological categories as CIN1, CIN2, and CIN3 [7], [8], [9]

The α-papillomavirus group includes high-risk HPV (HR-HPV) genotypes such as HPV16, 18, 31, 33, 35, 39, 45, 51, 52, 56, 58, 59, 66, 68, 73, and 82, which are carcinogenic. These genotypes are the primary cause of cervical and anal cancers, contributing to over 90% of the cases, as well as significant proportions of cancers in the vagina, vulva, penis, and head and neck. In most instances, HPV types 16 and 18 are responsible for 70% of cervical cancers, while 99.7% of cervical cancer cases being linked to persistent HR-HPV infection [10], [11], [12].

Globally, HR-HPV is responsible for approximately 5% of all cancers, affecting various sites including the cervix, oesophagus, bladder, oropharynx and others [13], [14], [15].

Cervical cancer ranks as the fourth most common cancer among women worldwide, with approximately 661,021 new cases and 348,189 deaths each year, predominantly affecting low- and middle-income countries (LMICs). In Tanzania, the female population is 31.7 million, with 14.5 million aged 15–49, a reproductive age group at higher risk for HPV due to sexual activity. Of the global cervical cancer cases, around 4115 occur in Tanzania, where at least 42.2% of women in this age group are diagnosed with cervical cancer [6], [16], [17].

HPV’s widespread impact goes beyond health, placing a substantial economic strain on healthcare systems due to prevention, diagnosis, and treatment costs. These economic and health burdens challenge communities, particularly in regions with limited access to healthcare [3], [4], [5].

Since 2014, the Tanzanian government, supported by global health organizations such as the World Health Organization (WHO), Gavi and Vaccine Alliance, has been implementing HPV vaccination programs. These initiatives began with pilot projects in the Kilimanjaro region and since then has expanded nationwide [15], [18].

The HPV vaccine roll out in Tanzania began with a successful pilot project in 2014, targeting girls aged 9 to 14 years. Building on this success, the Ministry of Health introduced a school based vaccination program in 2018, focusing on girls aged 14 years old. This initiative made notable progress in addressing HPV related health issues through extensive public health campaigns and broad vaccine distribution. The aim of vaccination was to prevent infections from the most common HR- HPV types and was implemented across various regions, primarily targeting young girls before they become sexually active [19], [20].

In 2018, Dr. Tedros Ghebreyesus, Director General of the World Health Organization (WHO), issued a call to action to eliminate cervical cancer as a public health problem. A central strategy in this initiative was for each country to prioritize the prevention of cervical cancer. In response, Tanzania launched a nationwide HPV vaccination program targeting 14 year old girls [21], [22], [23].

By the end of 2019, the program had achieved roughly 78% coverage for the first dose and 49% for the second dose, as of now 2024, coverage has improved to 79% for the first dose and 60% for the second dose. This progress highlights the continued need for secondary prevention efforts to reach 100% HPV vaccine coverage by 2030 [20], [24], [25], [26].

As stated by WHO [27], the elimination of cervical cancer requires reducing its occurence to fewer than 4 cases per 100,000 women. This goal can be met by implementing three essential strategies: ensuring 90% of girls are vaccinated by age 15, providing cervical screening for 70% of women at least twice by age 45, and treating 90% of women diagnosed with cervical pre-cancer or cancer. In response to the WHO’s recommendations on the importance of HPV vaccination programs, the Tanzania Ministry of Health launched an HPV vaccine campaign on April 22, 2024. The initiative aimed at immunizing more than five million girls aged 9 to 14 years and boost vaccine coverage beyond the current 79%. To date, over 600,000 girls in Tanzania have already received the HPV vaccine, demonstrating a thorough effort to increase vaccine uptake in alignment with WHO guidelines [17], [25], [28], [29].

Although effective HPV vaccines are available, their uptake is limited by age restrictions in vaccination programs. In Tanzania, these programs target preadolescent girls aged 9 to 14, leaving a broader age group unvaccinated. Those who do not receive the vaccine are at risk for HPV-related conditions, such as cervical pre-cancer and cancer, which necessitate treatment [17], [21], [23].

Like many other low- and middle-income countries (LMICs), Tanzania faces specific challenges in addressing the high prevalence of cervical cancer, including limited resources, inadequate healthcare infrastructure, and age restrictions to preventive measures like HPV vaccination and cervical screening.

The HPV vaccine in Tanzania highlighted the critical public health challenge posed by cervical cancer. A study by Baisley et al. [16] reported that more than 73% HPV prevalence among sexually active girls are aged 14 to 18, emphasizing the risk of persistent HR - HPV infections leading to cervical cancer, the leading cause of cancer mortality in Tanzania. The study recommended that early vaccination is crucial. The current programs focusing on 14 year old, press a need of expanding the age range that could significantly improve prevention efforts.

The study by Rosen et al. [23], highlighted the critical role of age in the uptake of the HPV vaccine, particularly within Tanzania’s school based vaccination program. The findings showed that early adolescence is a pivotal period for vaccine acceptance. Combining HPV vaccination with other programs targeting older age groups could potentially yield positive results in reducing HPV cases. However, challenges remain, especially in effectively reaching the targeted age group. Addressing these challenges is essential for improving HPV vaccine coverage in Tanzania.

Hsiao et al. [30], explored the cost implications of HPV vaccination in Tanzania, with a particular focus on vaccine uptake. They emphasized the considerable burden of cervical cancer in low resource settings, driven by high HPV infection rates. The study was in line with the World Health Organization’s recommendations for HPV vaccination, including the shift from a three dose to a two dose regimen as a strategy to enhance vaccine uptake by lowering costs and overcoming logistical challenges. Furthermore, the study reviewed recent studies suggesting that a single dose HPV vaccine might be as effective as multi dose regimens, potentially further reducing costs and improving uptake in Tanzania.

Nhumba and Sunguya [31], highlighted the critical issue of low uptake of the HPV vaccine, particularly the second dose, in Dar es Salaam, Tanzania. The study identified that only 21.3% of adolescent girls received the second dose, despite the availability of free vaccines. Factors such as age, awareness, and attitude towards the vaccine significantly influenced uptake. The study emphasized the need for enhanced community awareness and targeted interventions to improve vaccine coverage and reduce cervical cancer occurrence.

Mathematical modeling has become an important tool for studying the dynamics of HPV and designing effective control measures. Various mathematical models have been developed to investigate the dynamics of HPV transmission dynamics.

Rajan et al. [11], examined a mathematical model for understanding the dynamics of Human Papillomavirus (HPV) and its influence on the development of cervical cancer. The study provided a detailed exploration of how HPV, particularly HR-HPV strain, plays a critical role in the onset of cervical cancer. By focusing on the interactions between HPV infections and cervical cancer progression, the study highlighted the alarming fact that over 99.7% of cervical cancer cases are associated with HR-HPV infections. This strong correlation between HR-HPV and cervical cancer highlights the significance of these strains in driving the high occurrence of the disease.

Zhang et al. [32], presented a mathematical model to analyze the transmission dynamics of human papillomavirus focusing on the impact of vaccination and screening. The model considered various stages of HPV infection and the progression to cervical cancer, incorporating factors like vaccine efficacy, the necessity of re-vaccination, and the role of screening in controlling HPV spread. The results highlighted that increasing the vaccination rate is the most effective strategy to reduce $R_{e}$. Additionally, the findings suggested optimal approaches to minimize the costs associated with vaccination and screening while effectively managing HPV transmission.

Desta et al. [33] developed a mathematical model to study HPV transmission and its role in cervical cancer, with a focus on the impact of double-dose vaccination. The model categorizes the population into epidemiologically relevant compartments, including susceptible, vaccinated, and infected groups. Their analysis demonstrated that increasing vaccination coverage and recovery rates, combined with reducing HPV transmission through contact, can effectively reduce $R_{e}$ below unity, thereby lowering the prevalence of HPV.

This study by Drolet et al. [34] evaluated the potential impact of transitioning from a two-dose to a one-dose gender-neutral HPV vaccination program using a mathematical model. The analysis indicated that one-dose vaccination could prevent a comparable number of cervical cancers as the two-dose program across most scenarios of vaccine efficacy and protection duration. Even in pessimistic scenarios, one-dose vaccination is projected to be a more efficient use of vaccine doses while achieving cervical cancer elimination. If the protection from a single dose lasts beyond 25 years, it would safeguard individuals during their peak sexual activity years, thereby preventing the majority of HPV-related cancers. The study highlighted one-dose vaccination as an effective and efficient strategy, with the need for continuous monitoring of vaccine durability.

Cervical cancer, caused by Human Papillomavirus (HPV), remains a leading cause of cancer-related deaths among women in Sub-Saharan Africa, with Tanzania heavily affected. This study presents a mathematical model incorporating vaccination to evaluate its impact on HPV transmission, disease progression, and herd immunity.

## Model formulation

To comprehensively understand HPV transmission dynamics, a compartmental deterministic mathematical model was developed, focusing on three age groups: children aged 0–9, those aged 9–14, and individuals over 14. At any time t, children aged 0–9 and 9–14 are categorized into two groups: girls, denoted as G(t), and boys, denoted as B(t). The model also examines HPV transmission in a community implementing mass vaccination for girls aged 9–14 and newly sexually active females over 14, using a single dose of the quadrivalent Gardasil vaccine as suggested by [34], [35], [36], [37]. It assumes homogeneous mixing between sexually active males and females. It assumes homogeneous mixing between sexually active males and females. At any time t, the sexually active population is divided into female $N_{f}\left(t\right)$ and male $N_{m}\left(t\right)$ groups. The model accounts for total $N\left(t\right)=C\left(t\right)+G\left(t\right)+V_{1}\left(t\right)+B\left(t\right)+N_{f}\left(t\right)+N_{m}\left(t\right)$.

The female population is classified into five groups: susceptible females $S_{f}\left(t\right)$, vaccinated females $V_{f}\left(t\right)$, exposed females $E_{f}\left(t\right)$, infected females $I_{f}\left(t\right)$, females with cervical cancer Cc(t), and females who have recovered from cervical cancer $R_{c}\left(t\right)$. Similarly, the male population is divided into four classes: susceptible males $S_{m}\left(t\right)$, exposed males $E_{m}\left(t\right)$, infected males $I_{m}\left(t\right)$, and recovered males $R_{m}\left(t\right)$.

Children aged 0–9 are populated at a birth rate π and transition to boys or girls aged 9–14 at rates ξϵ and ξ(1−ϵ), respectively. All compartments experience natural mortality at a rate μ. Girls aged 9–14 increase through recruitment at rate (1−ϵ)ξ, vaccination at rate $\sigma_{1}$. Unvaccinated girls transition into the susceptible female group $S_{f}$ at rate $\xi \left(1-\sigma_{1}\right)$. Susceptible females either receive vaccination at rate $\sigma_{2}$ or become exposed to HPV through effective contact with infected males $I_{m}$.

Females above 14 can voluntarily receive vaccination, joining group $V_{2}$, transitioning either from $S_{f}$ at rate $\sigma_{2}$ or from $V_{1}$ due to aging at rate ξ. Non-vaccinated females acquire HPV at rate $\left(1-\sigma_{2}\right)\lambda_{1}$, entering the exposed class $E_{f}$. Exposed females transition to the infected class $I_{f}$ at rate $\gamma_{1}$. Infected females either recover at rate ρ or progress to cervical cancer at rate (1−ρ). Females with cervical cancer decrease due to disease-induced death at rate ν, while recovered females $R_{f}$ remain immune but decline naturally at rate μ.

The study assumes males are not vaccinated but serve as HPV carriers. Boys aged 9–14 transition to susceptible males $S_{m}$ at rate ξ and may acquire HPV through effective contact with infected females $I_{f}$ at rate $\lambda_{2}$. Exposed males $E_{m}$ develop clinical symptoms at rate $\gamma_{2}$, entering the infected class $I_{m}$. Infected males recover at rate κ, joining the recovered class $R_{m}$, which declines naturally at rate μ. The model does not include disease-induced deaths or cancer progression for males, reflecting the rarity of penile cancer.

To ensure clarity and biological accuracy, five assumptions were made to streamline the model, allowing for precise mathematical analysis and a better understanding of HPV dynamics.

• The administered vaccine has 100% efficacy against HR - HPV.
• Individuals under 14 are typically not sexually active and, therefore, are not at risk of contracting HR-HPV.
• Only female individuals are vaccinated.
• Recovered individual’s immunity does not wane with time.
• Sexually active females can contract HPV, caused by α-papillomavirus, which can lead to cervical for the female individuals.

**Fig. 1:**
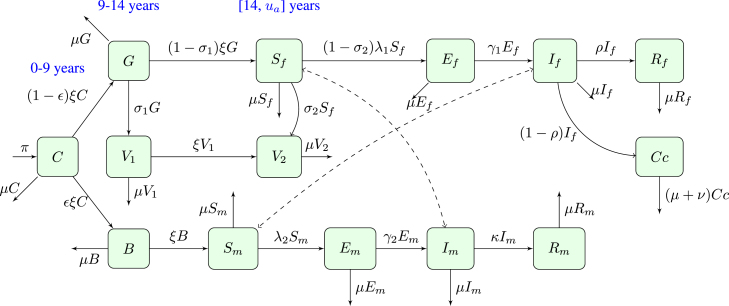
The schematic flowchart of HPV Vaccination model.

The model description and underlying assumptions led to the compartmental diagram presented in Fig. 1. The variables and parameters used to formulate the model are detailed in Table 1, Table 2, respectively. This is based on the relationships among the variables and parameters.

**Table 1:** Parameter description, value, and source.

Parameter	Description	Values	Source
π	Recruitment rate into children group	999,300	[38]
ϵ	Proportional of moving to 9−14 years age group	0.045	Calculated
$\sigma_{1}$$\left(\sigma_{2}\right)$	Vaccination rate for girls and (female)	0.016(0.87)	Calculated [39]
ξ	Transition rate from childhood to adulthood	0.32	[38]
κ(ρ)	Recovery rate for males (females)	0.85(0.15)	Calculated
μ	Natural mortality rate	0.015	[17], [38]
ν	Disease induced rate	0.01	[40]
$\beta_{m\to f}$$\left(\beta_{f\to m}\right)$	HPV transmission probability per contact for females (males)	0.557	[41]
$\gamma_{1}$$\left(\gamma_{2}\right)$	HPV transmission progression rate for females (males)	0.0157	[41]

**Table 2:** Description of the state variables.

Variable	Description	Variable	Description
C(t)	Children aged 0−9	G(t), B(t)	Girls and boys aged 9−14
$S_{f}\left(t\right)$, $S_{m}\left(t\right)$	Susceptible female and male aged [14, $u_{a}$]	$V_{1}\left(t\right)$, $V_{2}\left(t\right)$	Vaccinated female aged 9−14 and [14, $u_{a}$]
$I_{f}\left(t\right)$, $I_{m}\left(t\right)$	Infectious female and male aged [$14,u_{a}$]	Cc(t)	Female suffering from cervical cancer
$R_{f}\left(t\right)$, $R_{m}\left(t\right)$	Recovered female and male aged [14, $u_{a}$]	$\lambda_{1,2}$	Force of Infection

### Model equations

Fig. 1 illustrates the system of fourteen non-linear ordinary differential equations (ode) that describes the dynamics of HPV in presence of vaccination as given by:

(1) $$\left\{\begin{array}{ccc} \frac{dC}{dt} & = & \pi -\left(\mu +\xi \right)C,\\ \frac{dG}{dt} & = & \xi \left(1-\epsilon \right)C-\left(\mu +\sigma_{1}\right)G-\xi \left(1-\sigma_{1}\right)G,\\ \frac{dV_{1}}{dt} & = & \sigma_{1}G-\left(\mu +\xi \right)V_{1},\\ \frac{dS_{f}}{dt} & = & \xi \left(1-\sigma_{1}\right)G-\mu S_{f}-\sigma_{2}S_{f}-\left(1-\sigma_{2}\right)\lambda_{1}S_{f},\\ \frac{dV_{2}}{dt} & = & \sigma_{2}S_{f}+\xi V_{1}-\mu V_{2},\\ \frac{dE_{f}}{dt} & = & \left(1-\sigma_{2}\right)\lambda_{1}S_{f}-\left(\mu +\gamma_{1}\right)E_{f},\\ \frac{dI_{f}}{dt} & = & \gamma_{1}E_{f}-\left(\mu +\rho \right)I_{f},\\ \frac{dR_{f}}{dt} & = & \rho I_{f}-\mu R_{f},\\ \frac{dCc}{dt} & = & \left(1-\rho \right)I_{f}-\left(\mu +\nu \right)Cc,\\ \frac{dB}{dt} & = & \epsilon \xi C-\left(\mu +\xi \right)B,\\ \frac{dS_{m}}{dt} & = & \xi B-\mu S_{m}-\lambda_{2}S_{m},\\ \frac{dE_{m}}{dt} & = & \lambda_{2}S_{m}-\left(\mu +\gamma_{2}\right)E_{m},\\ \frac{dI_{m}}{dt} & = & \gamma_{2}E_{m}-\left(\mu +\kappa \right)I_{m},\\ \frac{dR_{m}}{dt} & = & \kappa I_{m}-\mu R_{m}. \end{array}\right)$$

where, $\lambda_{1}=\beta_{m\to f}\frac{I_{m}}{N_{m}}$, $\lambda_{2}=\beta_{f\to m}\frac{I_{f}}{N_{f}}$, and the corresponding initial conditions are C(0)≥0, G(0)≥0, $V_{1}\left(0\right)\geq 0$, $S_{f}\left(0\right)\geq 0$, $V_{2}\left(0\right)\geq 0$, $E_{f}\left(0\right)\geq 0$, $I_{f}\left(0\right)\geq 0$, $R_{f}\left(0\right)\geq 0$, Cc(0)≥0, B(0)≥0, $S_{m}\left(0\right)\geq 0$, $E_{m}\left(0\right)\geq 0$, $I_{m}\left(0\right)\geq 0$, $R_{m}\left(0\right)\geq 0$.

### Positivity of the solutions

For the model system (1) to be epidemiologically meaningful, it is vital to prove that all state variables are non-negative for all time. That means solutions of the model system (1) with non-negative initial conditions will remain non-negative for all t>0.

Theorem 1 *If the initial conditions {* C(0)≥0 *,* G(0)≥0 *,* $V_{1}\left(0\right)\geq 0$ *,* $S_{f}\left(0\right)\geq 0$ *,* $V_{2}\left(0\right)\geq 0$ *,* $E_{f}\left(0\right)\geq 0$ *,* $I_{f}\left(0\right)\geq 0$ *,* $R_{f}\left(0\right)\geq 0$ *,* Cc(0)≥0 *,* B(0)≥0 *,* $S_{m}\left(0\right)\geq 0$ *,* $E_{m}\left(0\right)\geq 0$ *,* $I_{m}\left(0\right)\geq 0$ *,* $R_{m}\left(0\right)\geq 0$ *} of the model system* (1) *are satisfied then the solutions {* C *,* G *,* $V_{1}$ *,* $S_{f}$ *,* $V_{2}$ *,* $E_{f}$ *,* $I_{f}$ *,* $R_{f}$ *,* Cc *,* B *,* $S_{m}$ *,* $E_{m}$ *,* $I_{m}$ *,* $R_{m}$ *} are non-negative for all* t≥0 *in* Ω *.*

Proof Considering the equations of the model system (1), then upon integration by separating the variables and applying initial conditions the positivity is proven as:

For children, it follows that,

(2) $$\frac{dC}{dt}=\pi -\left(\mu +\xi \right)C.$$

Upon separating the variables

$$\frac{dC}{dt}\geq -\left(\mu +\xi \right)C⟹\frac{dC}{C}\geq -\left(\mu +\xi \right)dt.$$

Applying anti-derivative with respect to time,

$\frac{dC}{C}\geq -\left(\mu +\xi \right)dt$ ⟹ $\int_{0}^{t}\frac{dC}{C}ds\geq \int_{0}^{t}-\left(\mu +\xi \right)ds$

$\ln C\left(t\right)-\ln C\left(0\right)\geq -\int_{0}^{t}\left(\mu +\xi \right)ds$ ⟹ $C\left(t\right)\geq C\left(0\right)e^{-\left(\mu +\xi \right)t}\geq 0.$

For girls, it follows that,

(3) $$\frac{dG}{dt}=\xi \left(1-\epsilon \right)C-\left(\mu +\sigma_{1}\right)G-\xi \left(1-\sigma_{1}\right)G.$$

Upon separating the variables

$$\frac{dG}{dt}\geq -\left(\mu +\sigma_{1}\right)G-\xi \left(1-\sigma_{1}\right)G⟹\frac{dG}{G}\geq -\left(\mu +\sigma_{1}+\xi \left(1-\sigma_{1}\right)\right)dt.$$

Applying anti-derivative with respect to time,

$\frac{dG}{G}\geq -\left(\mu +\sigma_{1}+\xi \left(1-\sigma_{1}\right)\right)dt$ ⟹ $\int_{0}^{t}\frac{dG}{G}ds\geq \int_{0}^{t}-\left(\mu +\sigma_{1}+\xi \left(1-\sigma_{1}\right)\right)ds$ $\ln G\left(t\right)-\ln G\left(0\right)\geq -\int_{0}^{t}\mu ds$ ⟹ $G\left(t\right)\geq G\left(0\right)e^{-\left(\mu +\sigma_{1}+\xi \left(1-\sigma_{1}\right)\right)t}\geq 0.$

For susceptible female, it follows that,

(4) $$\frac{dS_{f}}{dt}=\xi \left(1-\sigma_{1}\right)G-\mu S_{f}-\sigma_{2}S_{f}-\left(1-\sigma_{2}\right)\lambda_{1}S_{f}.$$

Upon separating the variables$\frac{dS_{f}}{dt}\geq -\left(\mu +\sigma_{2}+\left(1-\sigma_{2}\right)\lambda_{1}\right)S_{f}$ ⟹ $\frac{dS_{f}}{S_{f}}\geq -\left(\mu +\sigma_{2}+\left(1-\sigma_{2}\right)\lambda_{1}\right)dt$

Applying anti-derivative with respect to time, $\frac{dS_{f}}{S_{f}}\geq -\left(\mu +\sigma_{2}+\left(1-\sigma_{2}\right)\lambda_{1}\right)dt$ ⟹ $\int_{0}^{t}\frac{dS_{f}}{S_{f}}ds\geq \int_{0}^{t}-\left(\mu +\sigma_{2}+\left(1-\sigma_{2}\right)\lambda_{1}\right)ds$

$\ln S_{f}\left(t\right)-\ln S_{f}\left(0\right)\geq -\int_{0}^{t}\left(\mu +\sigma_{2}+\left(1-\sigma_{2}\right)\lambda_{1}\right)ds$,⟹$S_{f}\left(t\right)\geq S_{f}\left(0\right)e^{-\int_{0}^{t}\left(\mu +\sigma_{2}+\left(1-\sigma_{2}\right)\lambda_{1}\right)ds}\geq 0.$

Similarly, the proof of the non-negativity of the remaining functions is done recursively, demonstrating the positivity for $V_{1}$, $V_{2}$, $E_{f}$, $I_{f}$, $R_{f}$, Cc, B, $S_{m}$, $I_{m}$, and $R_{m}$ in the model system (1).

(5) $$\begin{array}{ccc} C\left(t\right) & \geq & C\left(0\right)e^{-\left(\mu +\xi \right)t}\geq 0,\\ G\left(t\right) & \geq & G\left(0\right)e^{-\left(\mu +\sigma_{1}+\xi \left(1-\sigma_{1}\right)\right)t}\geq 0,\\ V_{1}\left(t\right) & \geq & V_{1}\left(0\right)e^{-\left(\mu +\xi \right)t}\geq 0,\\ S_{f}\left(t\right) & \geq & S_{f}\left(0\right)e^{-\int_{0}^{t}\left(\mu +\sigma_{2}+\left(1-\sigma_{2}\right)\lambda_{1}\right)ds}\geq 0,\\ V_{2}\left(t\right) & \geq & V_{2}\left(0\right)e^{-\mu t}\geq 0,\\ E_{f}\left(t\right) & \geq & E_{f}\left(0\right)e^{-\left(\mu +\gamma_{1}\right)t}\geq 0,\\ I_{f}\left(t\right) & \geq & I_{f}\left(0\right)e^{-\left(\mu +\rho \right)t}\geq 0,\\ Cc\left(t\right) & \geq & Cc\left(0\right)e^{-\left(\mu +\nu \right)t}\geq 0,\\ R_{f}\left(t\right) & \geq & R_{f}\left(0\right)e^{-\mu t}\geq 0,\\ B\left(t\right) & \geq & B\left(0\right)e^{-\left(\mu +\xi \right)t}\geq 0,\\ S_{m}\left(t\right) & \geq & S_{m}\left(0\right)e^{-\int_{0}^{t}\left(\mu +\lambda_{2}\right)ds}\geq 0,\\ E_{m}\left(t\right) & \geq & E_{m}\left(0\right)e^{-\left(\mu +\gamma_{2}\right)t}\geq 0,\\ I_{m}\left(t\right) & \geq & I_{m}\left(0\right)e^{-\left(\mu +\kappa \right)t}\geq 0,\\ R_{m}\left(t\right) & \geq & R_{m}\left(0\right)e^{-\mu t}\geq 0. \end{array}$$

Hence, the solution set {C, G, $V_{1}$, $S_{f}$, $V_{2}$, $E_{f}$, $I_{f}$, $R_{f}$, Cc, B, $S_{m}$, $E_{m}$, $I_{m}$, $R_{m}$} of the model system (1) as the Eqs. (5) illustrates non-negative for all t≥0 in Ω. □

### Invariant region

The invariant region shows the domain in which the HPV-vaccine solution model (1) is mathematically and biologically plausible. From the model, all state variables and model parameters are non-negative for all t≥0.

Theorem 2 *All solution set of the model system* (1) *with initial solutions* C(0)≥0 *,* G(0)≥0 *,* $V_{1}\left(0\right)\geq 0$ *,* $S_{f}\left(0\right)\geq 0$ *,* $V_{2}\left(0\right)\geq 0$ *,* $E_{f}\left(0\right)\geq 0$ *,* $I_{f}\left(0\right)\geq 0$ *,* $R_{f}\left(0\right)\geq 0$ *,* Cc(0)≥0 *,* B(0)≥0 *,* $S_{m}\left(0\right)\geq 0$ *,* $E_{m}\left(0\right)\geq 0$ *,* $I_{m}\left(0\right)\geq 0$ *and* $R_{m}\left(0\right)\geq 0$ *, defined in the region* Ω *in* $R_{+}^{14}$ *where*

$\Omega =\{C,B,G,V_{1},V_{2},S_{f},E_{f},I_{f},R_{f},Cc,S_{m},E_{m},I_{m},R_{m}\in R_{+}^{14}$ $:N\left(t\right)\leq \frac{\pi }{\mu }\}$ *is positively bounded.*

Proof For all positive initial conditions C(0)≥0, G(0)≥0, $V_{1}\left(0\right)\geq 0$, $S_{f}\left(0\right)\geq 0$, $V_{2}\left(0\right)\geq 0$, $E_{f}\left(0\right)\geq 0$, $I_{f}\left(0\right)\geq 0$, $R_{f}\left(0\right)\geq 0$, Cc(0)≥0, B(0)≥0, $S_{m}\left(0\right)\geq 0$, $E_{m}\left(0\right)\geq 0$, $I_{m}\left(0\right)\geq 0$ and $R_{m}\left(0\right)\geq 0$ it is necessary to prove that the solution set {C, G, $S_{f}$, $E_{f}$, $V_{1}$, $V_{2}$, $I_{f}$, $R_{f}$, Cc, B, $S_{m}$, $E_{m}$, $I_{m}$, $R_{m}$} $\in R_{+}^{14}$ is positive bounded. Then, consider the total population given by;

(6) $$\frac{dN}{dt}=\pi -\mu N-\left(\sigma_{1}G+V_{1}+B\right)\xi \leq \pi -\mu N,$$

Then,

(7) $$⟹N\left(t\right)\leq \frac{\pi }{\mu }+\left(N\left(0\right)-\frac{\pi }{\mu }\right)e^{-\mu t}.$$

Eq. (7) show that the model system (1) is epidemiologically meaningful and particularly if all the state variables are positive. Since, the differential inequality $\frac{dN}{dt}\leq \pi -\mu N$ has the solution of the form $0\leq N\left(t\right)\leq \frac{\pi }{\mu }$ as t→∞. Therefore, the solution of the system (1) is bounded and it attracts all solutions in the domain Ω. Hence, it is sufficient to study the dynamics of the model system (1) in Ω. □

#### Disease free equilibrium point (DFE)

The disease-free equilibrium point (DFE) denoted by $E_{0}=(C^{0}$, $G^{0}$, $V_{1}^{0}$, $S_{f}^{0}$, $V_{2}^{0}$, $E_{f}^{0}$, $I_{f}^{0}$, $R_{f}^{0}$, $Cc^{0}$, $B^{0}$, $S_{m}^{0}$, $E_{m}^{0}$, $I_{m}^{0}$, $R_{m}^{0})$ of the model system (1) is $E_{0}=\Gamma_{1}⨁\Gamma_{2}⨁\Gamma_{3}$, where $\Gamma_{1}=(C^{0}$, $G^{0}$, $V_{1}^{0})$, $\Gamma_{2}=(S_{f}^{0}$, $V_{2}^{0})$ and $\Gamma_{3}=(E_{f}^{0}$, $I_{f}^{0}$, $R_{f}^{0}$, $Cc^{0}$, $B^{0}$, $S_{m}^{0}$, $E_{m}^{0}$, $I_{m}^{0}$, $R_{m}^{0})$ such that

$$\Gamma_{1}=\;\left[\frac{\pi }{\mu +\xi },\;\;\frac{\left(1-ɛ\right)\pi \xi }{{\left(\mu +\xi \right)}^{2}+\left(\mu +\xi \right)\left(1-\xi \right)\sigma_{1}},\;\;\frac{\left(1-ɛ\right)\pi \xi \sigma_{1}}{{\left(\mu +\xi \right)}^{2}+\left(\mu +\xi \right)\left(1-\xi \right)\sigma_{1}}\right]$$

$$\Gamma_{2}=\;\left[\frac{\left(1-\sigma_{1}\right)\left(1-ɛ\right)\pi \xi^{2}}{\left({\left(\mu +\xi \right)}^{3}+{\left(\mu +\xi \right)}^{2}\left(1-\xi \right)\sigma_{1}\right)\left(\mu +\sigma_{2}\right)},\;\;\frac{\left(1-ɛ\right)\xi }{\mu \left(\mu +\sigma_{2}\right){\left(\mu +\xi \right)}^{2}}\left(\frac{\left(\mu +\sigma_{2}\right)\sigma_{1}+\pi \xi }{\left(\mu +\xi \right)+\left(1-\xi \right)\sigma_{1}}\right)\right]$$

$$\Gamma_{3}=\;\left[0,\;\;0,\;\;0,\;\;0,\;\;\frac{\pi \xi }{{\left(\mu +\xi \right)}^{2}},\;\;\frac{\pi \xi^{2}}{\mu {\left(\mu +\xi \right)}^{2}},\;\;0,\;\;0,\;\;0\right]$$

### Reproduction number of HPV

In the context of medicine and epidemiology, when control measures are implemented, the parameter used to assess how effectively the disease is kept under control is referred to as the effective reproduction number, as explained in the work by [42]. The reproduction number, $R_{e}$, represents the mean number of secondary cases generated by a single infected individual during their entire period of infectiousness. It serves as a threshold to assess whether an HPV infection will fade out or persist. When $R_{e}\leq 1$, each infectious individual transmits the virus to fewer than one person, leading to a decline in transmission over time. Conversely, when $R_{e}>1$, the infection spreads as each infectious individual infects more than one person, potentially increasing HPV cases and, subsequently, cervical cancer incidence in the female population. The Graph-Theoretic (GT) method, as introduced by [43], [44], [45], was used to compute the threshold parameter ($R_{e}$) through a GT-based Gaussian elimination technique, as per Theorem 3.

Theorem 3 *Let F be a non-zero, non-negative matrix, and V a non-singular M-matrix, with the matrix* F−V *assumed to be irreducible. Then, the reproduction number* $R_{e}=\rho \left(FV^{-1}\right)>0$ *, and* $R_{e}$ *is the reciprocal of the smallest positive root* x *of the polynomial equation* det(Fx−V)=0.

Proof Given the assumptions, $FV^{-1}$ is a non-zero, non-negative matrix. According to Li and Schneider [46], the principal submatrix corresponding to the non-zero rows of F is irreducible. Therefore, $\lambda =\rho \left(FV^{-1}\right)>0$ is the largest positive root of the polynomial equation $\det\left(\lambda I-FV^{-1}\right)=0.$ Since V is non-singular and λ>0, this equation is equivalent to $\det\left(F\lambda^{-1}-V\right)=0,$ where $V-F\lambda^{-1}$ is a singular M-matrix. The reciprocal $\left(\lambda^{-1}\right)$ is the smallest positive root. From the definition of $R_{e}=\rho \left(FV^{-1}\right)$. □

The method begins by differentiating new infections from all other changes occurring in the population. Then, F is n×n matrix and V is m×m non-singular matrix which is determined by partial differentiation and evaluating it at equilibrium point. Matrices F and V are obtained as follows:

(8) $$F={\left[\frac{\partial F_{i}}{\partial \left(E_{f},I_{f},Cc,E_{m},I_{m}\right)}\right]}_{E_{0}}\text{and}\;\;V={\left[\frac{\partial V_{i}}{\partial \left(E_{f},I_{f},Cc,E_{m},I_{m}\right)}\right]}_{E_{0}}$$

As [43], [44], [45], [46] observed, the GT method factors in only the infected compartments. Meanwhile, the model system (1), the infectious compartments are

(9) $$\left\{\begin{array}{ccc} \frac{dE_{f}}{dt} & = & \left(1-\sigma_{2}\right)\lambda_{1}S_{f}-\left(\mu +\gamma_{1}\right)E_{f},\\ \frac{dI_{f}}{dt} & = & \gamma_{1}E_{f}-\left(\mu +\rho \right)I_{f},\\ \frac{dCc}{dt} & = & \left(1-\rho \right)I_{f}-\left(\mu +\nu \right)Cc,\\ \frac{dE_{m}}{dt} & = & \lambda_{2}S_{m}-\left(\mu +\gamma_{2}\right)E_{m},\\ \frac{dI_{m}}{dt} & = & \gamma_{2}E_{m}-\left(\mu +\kappa \right)I_{m}. \end{array}\right)$$

Now, from (9) $F_{i}$ and $V_{i}$ are as follows

(10) $$F_{i}=\left(\begin{array}{c} F_{1}\\ F_{2}\\ F_{3}\\ F_{4}\\ F_{5} \end{array}\right)=\left(\begin{array}{c} \left(1-\sigma_{2}\right)\lambda_{1}S_{f}\\ 0\\ 0\\ \lambda_{2}S_{m}\\ 0 \end{array}\right)=\left(\begin{array}{c} \left(1-\sigma_{2}\right)\beta_{m\to f}\frac{I_{m}}{N_{m}}S_{f}\\ 0\\ 0\\ \beta_{f\to m}\frac{I_{f}}{N_{f}}S_{m}\\ 0 \end{array}\right),$$

and

(11) $$V_{i}=\left(\begin{array}{c} V_{1}\\ V_{2}\\ V_{3}\\ V_{4}\\ V_{5} \end{array}\right)=\left(\begin{array}{c} \left(\mu +\gamma_{1}\right)E_{f}\\ \left(\mu +\rho \right)I_{f}-\gamma_{1}E_{f}\\ \left(\mu +\nu \right)Cc-\left(1-\rho \right)I_{f}\\ \left(\mu +\gamma_{2}\right)E_{m}\\ \left(\mu +\kappa \right)I_{m}-\gamma_{2}E_{m} \end{array}\right).$$

Now, evaluating the partial derivative of $F_{i}$ and $V_{i}$ at DFE with respect to $E_{f},I_{f},Cc,E_{m}$ and $I_{m}$ gives F and V, that is then evaluated to obtain the matrix $H=F\lambda^{-1}-V$ as follows:

(12) $$H=\left(\begin{array}{ccccc} -\left(\mu +\gamma_{1}\right) & 0 & 0 & 0 & \frac{\left(1-\sigma_{2}\right)\beta_{m\to f}S_{f}^{0}}{N_{m}^{0}}\lambda^{-1}\\ \gamma_{1} & -\left(\mu +\rho \right) & 0 & 0 & 0\\ 0 & \left(1-\rho \right) & -\left(\mu +\nu \right) & 0 & 0\\ 0 & 0 & \frac{\beta_{f\to m}S_{m}^{0}}{N_{f}^{0}}\lambda^{-1} & -\left(\mu +\gamma_{2}\right) & 0\\ 0 & 0 & 0 & \gamma_{2} & -\left(\mu +\kappa \right) \end{array}\right).$$

This matrix corresponds to a system of interactions between different compartments of a model, where each entry represents the transition rate between states. The digraph representation of $F\lambda^{-1}-V$ translates these matrix interactions into a visual form, where nodes represent states, and directed edges capture the transition rates between states as specified by the matrix entries.

#### Matrix and digraph relationship

In the digraph 2 representation, nodes $E_{f}$, $I_{f}$, Cc, $E_{m}$, and $I_{m}$ correspond to distinct states. The directed edges between these nodes, labeled with transition rates such as $\gamma_{1}$ and $\frac{\left(1-\sigma_{2}\right)\beta_{m\to f}S_{f}^{0}}{N_{m}^{0}}\lambda^{-1}$, reflect the non-zero matrix elements that describe transition rates. Loops on the nodes indicate self-transitions, representing diagonal elements like $-\left(\mu +\gamma_{1}\right)$, which correspond to natural death and exit rate from the compartments.

These diagonal elements appear as loops in the digraph, illustrating the rates at which the system exits from each state. Off-diagonal elements signify transitions between different nodes; for instance, the term $\gamma_{1}$ connecting $E_{f}\to I_{f}$ represents an edge from $E_{f}$ to $I_{f}$ in the digraph, symbolizing the flow from exposed to infectious individuals.

**Fig. 2:**
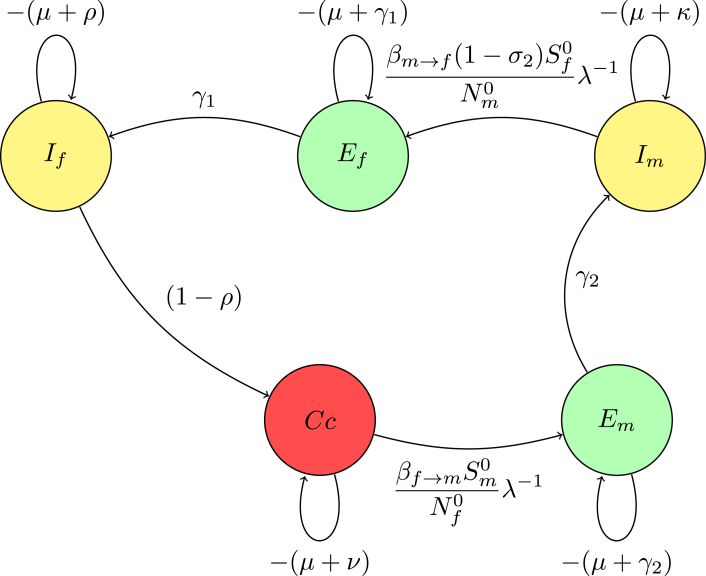
(A*) Digraph representation of $F\lambda^{-1}-V$.

#### Digraph reduction and Gaussian elimination

According to Theorem 3, digraph reduction is analogous to the process of Gaussian elimination on the matrix $F\lambda^{-1}-V$. In Gaussian elimination, the matrix is systematically simplified by eliminating non-diagonal elements, much like how digraph reduction removes intermediate nodes by adjusting the weights of the connecting edges until a trivial node remains. A trivial node is characterized by a self-loop with a weight of −1 representing self-regulating processes or constant death rate. The reduction of the digraph is carried out in accordance with specific rules.

**Rule 1.** In the matrix, creating a trivial node is achieved by normalizing the diagonal entry for that node, which involves dividing all incoming edges by this value. In Gaussian elimination, this process corresponds to normalizing a row.

**Rule 2.** Eliminating arcs through a trivial node reduces the graph by collapsing paths through the node into direct connections between the remaining nodes. In Gaussian elimination, this is analogous to removing elements from rows, simplifying the system without altering the determinant.

**Fig. 3:**
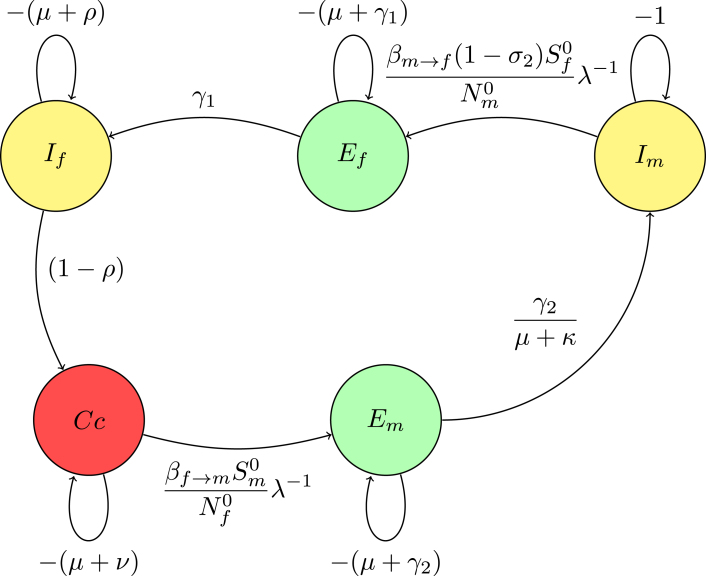
(**A**) Creating a trivial node at node $I_{m}$.

Reduction on digraph associated with node $I_{m}$, convert a loop with weight −(μ+κ)<0 at node $I_{m}$ to −1, divide the weights of all incoming arcs to node $I_{m}$ by μ+κ. This operation creates a trivial node $I_{m}$ with a loop weight of −1. Mathematically, this corresponds to dividing the row for $I_{m}$ in the determinant $\det\left(F\lambda^{-1}-V\right)$ by μ+κ, effectively scaling the determinant by $\frac{1}{\mu +\kappa }$. For a trivial node $I_{m}$ on the path $E_{m}\to I_{m}$, replace the arc $E_{m}\to E_{f}$ with a single arc, weighted by $\frac{\gamma_{2}}{\mu +\kappa }$. If node $I_{m}$ has no incoming or outgoing paths, it can be ignored. The accompanying Fig. 3 demonstrates the process of creating a trivial node at $I_{m}$ and the corresponding connections (arcs) involved in this transformation. This visual representation aids in understanding how the weights and connections in the digraph change as a result of applying the Gaussian elimination algorithm.

The associated matrix is presented

(13) $$M_{1}=\left(\begin{array}{cccc} -\left(\mu +\gamma_{1}\right) & 0 & 0 & \frac{\left(1-\sigma_{2}\right)\gamma_{2}\beta_{m\to f}S_{f}^{0}}{N_{m}^{0}\left(\mu +\kappa \right)}\lambda^{-1}\\ \gamma_{1} & -\left(\mu +\rho \right) & 0 & 0\\ 0 & \left(1-\rho \right) & -\left(\mu +\nu \right) & 0\\ 0 & 0 & \frac{\beta_{f\to m}S_{m}^{0}}{N_{f}^{0}}\lambda^{-1} & -\left(\mu +\gamma_{2}\right) \end{array}\right).$$

**Fig. 4:**
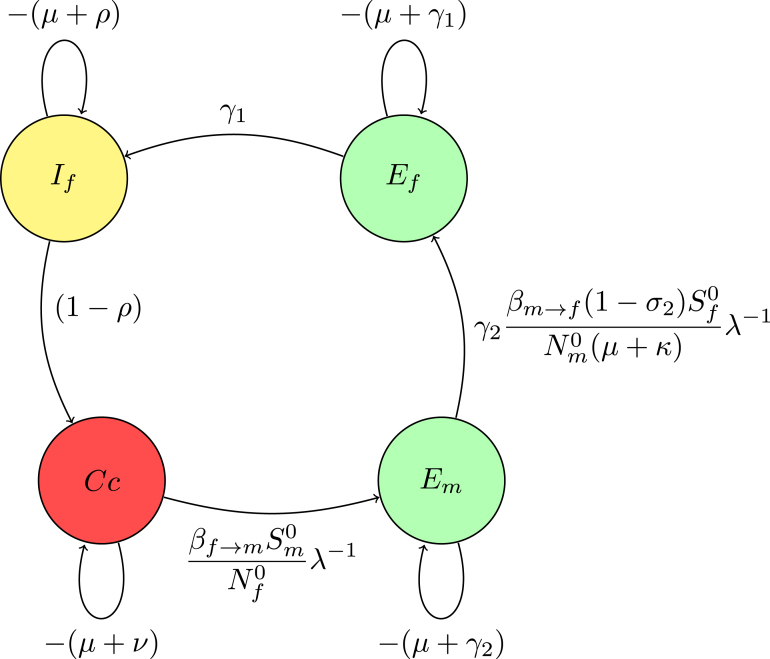
(B*) Digraph representation without node $I_{m}$.

Reduction on digraph associated with node $E_{m}$ convert a loop with weight $-\left(\mu +\gamma_{2}\right)<0$ at node $E_{m}$ to −1, divide the weights of all incoming arcs to node $E_{m}$ by $\mu +\gamma_{2}$. This operation creates a trivial node $E_{m}$ with a loop weight of −1.

For a trivial node $E_{m}$ on the path $E_{m}\to E_{f}$, replace the arc $Cc\to E_{f}$ weighted by the product of the original arc weight and the weight of the arc $Cc\to E_{m}$, $\frac{\beta_{f\to m}S_{m}^{0}}{N_{f}^{0}\left(\mu +\gamma_{2}\right)}\lambda^{-1}$.

The associated matrix of reduction on digraph without node $E_{m}$

(14) $$M_{2}=\left(\begin{array}{ccc} -\left(\mu +\gamma_{1}\right) & 0 & \frac{\beta_{f\to m}S_{m}^{0}\left(1-\sigma_{2}\right)\beta_{m\to f}S_{f}^{0}}{N_{f}^{0}N_{m}^{0}\left(\mu +\kappa \right)\left(\mu +\gamma_{2}\right)}\lambda^{-2}\\ \gamma_{1} & -\left(\mu +\rho \right) & 0\\ 0 & \left(1-\rho \right) & -\left(\mu +\nu \right) \end{array}\right).$$

**Fig. 5:**
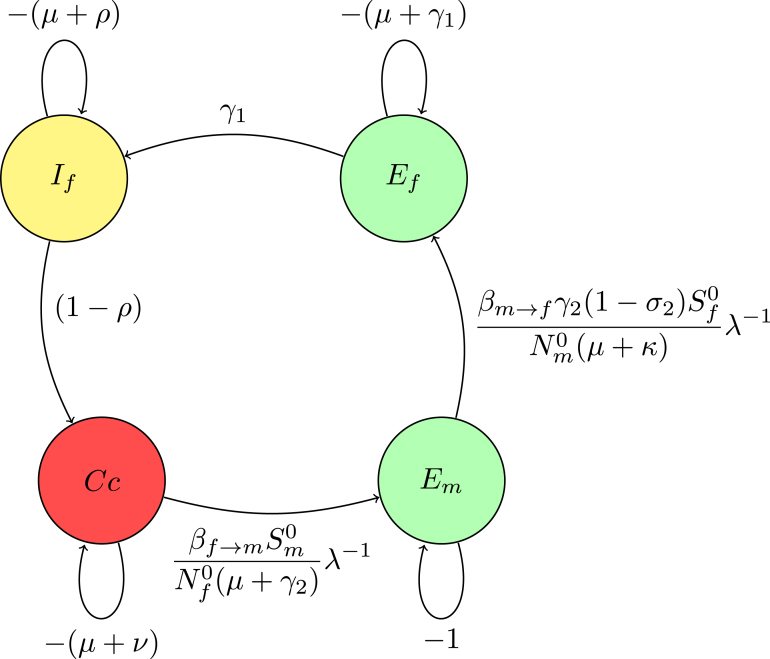
(**B**) Creating a trivial node at node $E_{m}$.

**Fig. 6:**
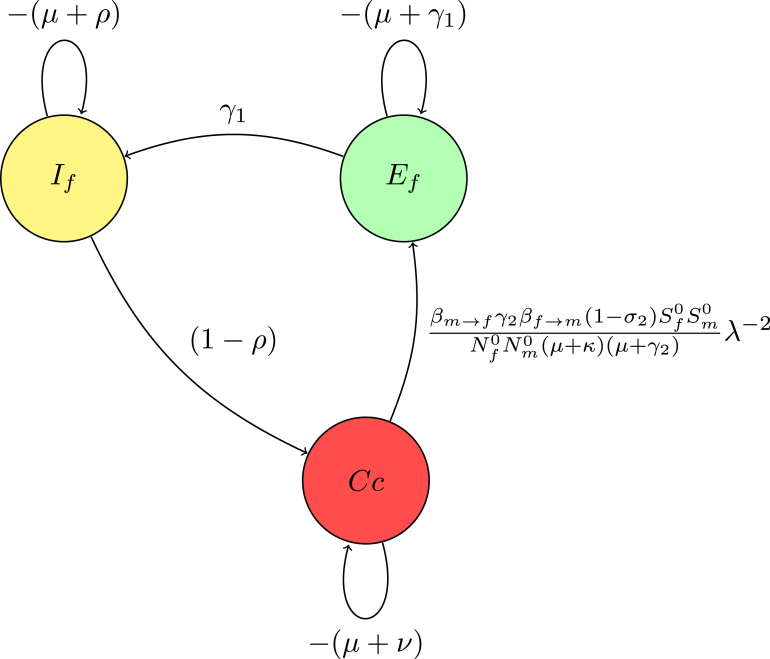
(C*) Digraph Representation without node $E_{m}$.

The Gaussian algorithm for all other nodes in Fig. 8, Fig. 9, Fig. 10 is performed in the same manner as shown in Fig. 5, Fig. 6 to yield the result (see Fig. 10).

The associated matrix of reduction on digraph without node Cc

(15) $$M_{3}=\left(\begin{array}{cc} -\left(\mu +\gamma_{1}\right) & \frac{\beta_{f\to m}S_{m}^{0}\left(1-\sigma_{2}\right)\left(1-\rho \right)\beta_{m\to f}S_{f}^{0}}{N_{f}^{0}N_{m}^{0}\left(\mu +\kappa \right)\left(\mu +\gamma_{2}\right)\left(\mu +\nu \right)}\lambda^{-2}\\ \gamma_{1} & -\left(\mu +\rho \right) \end{array}\right).$$

According to Theorem 3, setting the weight of the loop at node $E_{f}$ to zero yields the polynomial equation $\lambda^{-2}$. After simplification and substituting the values of $N_{f}^{0}$, $N_{m}^{0}$, $S_{f}^{0}$, and $S_{m}^{0}$ into the equation, the reciprocal of the smallest positive root is the expression for $R_{e}$,

(16) $$\lambda^{2}=\frac{\left(1-\rho \right)\beta_{m\to f}\gamma_{2}\gamma_{1}\beta_{f\to m}\left(1-\sigma_{2}\right)S_{f}^{0}S_{m}^{0}}{\left(\mu +\kappa \right)\left(\mu +\gamma_{2}\right)\left(\mu +\nu \right)\left(\mu +\rho \right)\left(\mu +\gamma_{1}\right)N_{f}^{0}N_{m}^{0}}$$

$$⟹R_{e}=\sqrt{\frac{\pi \gamma_{2}\gamma_{1}\mu^{2}\left(1-\sigma_{2}\right)\left(1-\sigma_{1}\right)\left(1-\rho \right)\beta_{f\to m}\beta_{m\to f}}{\left(\mu +\kappa \right)\left(\mu +\gamma_{2}\right)\left(\mu +\nu \right)\left(\mu +\rho \right)\left(\mu +\gamma_{1}\right)\left[\left(1-ɛ\right)\xi^{2}+\left(\mu +\sigma_{2}\right)\sigma_{1}\right]}}$$

### Global stability at the disease - free equilibrium point

This part explains that when the $R_{e}\leq 1$, HPV infections have the potential to be eliminated from the community, regardless of the initial size.

Theorem 4 *Assume that the matrix* H *representing the transmission dynamics of the disease, is irreducible. Then, the disease-free equilibrium is globally asymptotically stable within the feasible region* Ω *for* $R_{e}\leq 1$ *otherwise unstable.*

Proof Let C,A=(G,B), $V=\left(V_{1},V_{2}\right)$, $S=\left(S_{f},S_{m}\right)$, $S_{0}=\left(S_{f}^{0},S_{m}^{0}\right)$, $E=\left(E_{f},E_{m}\right)$, $I=\left(I_{f},I_{m}\right)$, Cc and $R=\left(R_{f},R_{m}\right)$ be the column vectors. Let X=(E,Cc,I) and Y=(C,A,V,S,R) be the column vectors representing the infectious and non-infectious states, respectively.

We define a vector function

$$g_{1}\left(X,Y\right)=\left(F-V\right)\left(X\right)-F\left(X,Y\right)+V\left(X,Y\right).$$

Here, the matrices F, V, F, and V are explained in Eqs. (8) – (11), respectively that basically form system (1) as

(17) $$\left\{\begin{array}{ccc} Y^{\prime } & = & g_{2}\left(X,Y\right),\\ X^{\prime } & = & \left(F-V\right)X+g_{1}\left(X,Y\right),\;\;\;\text{where}\;g_{1}\left(0,Y\right)=0\;\text{and}\;g_{2}\left(X,Y\right)=0. \end{array}\right)$$

In the feasible region Ω, it follows that $\;g_{2}\left(X,Y\right)=0$. The disease-free state $\left(0,Y^{0}\right)$ of system (17) is equivalent to $E_{0}$ for system (1).

Since the digraph $G\left(A^{*}\right)$ is strongly connected, that means an oriented path connects any two non-intersecting vertices. Therefore, the matrix H is non-negative and irreducible. From the Perron–Frobenius theorem [46], there exists a non-negative left eigenvector, say $\omega^{T}$ corresponding to the unique largest real eigenvalue of ρ(H).

For system (17), let the Lyapunov function (18) be on the feasible region Ω

(18) $$L=\omega^{T}V^{-1}X.$$

On differentiating L, and substituting the solutions of system (17), it is obtained that

(19) $$L^{\prime }=\omega^{T}V^{-1}X^{\prime }$$

$$=\omega^{T}V^{-1}\left(\left(F-V\right)X+g_{1}\left(X,Y\right)\right)=\left(\rho \left(H\right)-1\right)\omega^{T}X+\omega^{T}V^{-1}g_{1}\left(X,Y\right),\;\;\;\text{where}\;\rho \left(H\right)=R_{e}.$$

It is biologically observed that if $R_{e}\leq 1$, then $L^{\prime }\leq 0$ since $g_{2}\left(X,Y\right)$ is zero in the region Ω and $L^{\prime }=0$ if and only if $\omega^{T}X=0$, which implies X=0 (DFE). Now, it is concluded that the singleton $\left\{E_{0}\right\}$ is the invariant set in Ω where $L^{\prime }=0$. Consequently, by LaSalle’s invariance principle [47], $E_{0}$ is globally asymptotically stable in Ω for $R_{e}\leq 1$.

If $R_{e}>1$ along with the conditions X>0 and $Y=Y_{0}$, it gives $L^{\prime }>0$ in a small arbitrary neighborhood of $E_{0}$ in $\Omega^{0}$ (interior of Ω). Therefore, the disease-free equilibrium $E_{0}$ is unstable for $R_{e}>1$.

As a consequence of the result above, we can confidently deduce that HPV can be eradicated from the host community if the value of $R_{e}$ is reduced and maintained below unity. Fig. 11 illustrates the validation of the global stability analysis for the disease-free equilibrium point. □

### Endemic equilibrium point

#### Existence of the equilibrium solutions

The endemic equilibrium $E^{*}$ represents the state where HPV infections within the population. It is determined by equating the right-hand side expressions of the model system (1) to zero and solving for the state variables. Therefore, the endemic equilibrium is expressed as $E^{*}=(C^{*}$, $G^{*}$,$V_{1}^{*}$,$S_{f}^{*}$, $V_{2}^{*}$, $E_{f}^{*}$, $I_{f}^{*}$, $R_{f}^{*}$, $Cc^{*}$, $B^{*}$, $S_{m}^{*}$, $I_{m}^{*}$, and $R_{m}^{*})$

(20) $$\left\{\begin{array}{ccc} C^{*} & = & \frac{\pi }{\mu +\xi },\\ G^{*} & = & \frac{\left(1-ɛ\right)\pi \xi }{{\left(\mu +\xi \right)}^{2}+\left(\mu +\xi \right)\left(1-\xi \right)\sigma_{1}},\\ V_{1}^{*} & = & \frac{\left(1-ɛ\right)\pi \xi \sigma_{1}}{{\left(\mu +\xi \right)}^{2}+\left(\mu +\xi \right)\left(1-\xi \right)\sigma_{1}},\\ S_{f}^{*} & = & \frac{\pi \xi^{2}\left(1-\sigma_{1}\right)\left(1-\epsilon \right)}{\left(\mu +\xi \right)\left(\left(1-\sigma_{2}\right)\lambda_{1}^{*}+\mu +\sigma_{2}\right)\left(\mu +\sigma_{1}+\left(1-\sigma_{1}\right)\xi \right)},\\ V_{2}^{*} & = & \frac{\pi \xi^{2}\left(1-\epsilon \right)\left(\left(1-\sigma_{1}\right)\lambda_{1}^{*}\sigma_{2}+\left(1-\sigma_{2}\right)\sigma_{1}\mu +\left(1-\sigma_{1}\right)\xi \sigma_{2}+\sigma_{2}\sigma_{1}\right)}{\mu {\left(\mu +\xi \right)}^{2}\left(\left(1-\sigma_{2}\right)\lambda_{1}^{*}+\mu +\sigma_{2}\right)\left(\mu +\sigma_{1}+\left(1-\sigma_{1}\right)\xi \right)},\\ E_{f}^{*} & = & \frac{\pi \lambda_{1}^{*}\xi^{2}\left(1-\sigma_{1}\right)\left(1-\sigma_{2}\right)\left(1-\epsilon \right)}{\mu \left(\gamma_{1}+\mu \right)\left(\mu +\xi \right)\left(\left(1-\sigma_{2}\right)\lambda_{1}^{*}+\mu +\sigma_{2}\right)},\\ I_{f}^{*} & = & \frac{\pi \gamma_{1}\lambda_{1}^{*}\xi^{2}\left(1-\sigma_{1}\right)\left(1-\sigma_{2}\right)\left(1-\epsilon \right)}{\mu \left(\gamma_{1}+\mu \right)\left(\mu +\xi \right)\left(\mu +\rho \right)\left(\left(1-\sigma_{2}\right)\lambda_{1}^{*}+\mu +\sigma_{2}\right)},\\ R_{f}^{*} & = & \frac{\pi \gamma_{1}\lambda_{1}^{*}\xi^{2}\rho \left(1-\sigma_{1}\right)\left(1-\sigma_{2}\right)\left(1-\epsilon \right)}{\mu^{2}\left(\gamma_{1}+\mu \right)\left(\mu +\xi \right)\left(\mu +\rho \right)\left(\left(1-\sigma_{2}\right)\lambda_{1}^{*}+\mu +\sigma_{2}\right)},\\ Cc^{*} & = & \frac{\pi \gamma_{1}\lambda_{1}^{*}\xi^{2}\left(1-\rho \right)\left(1-\sigma_{1}\right)\left(1-\sigma_{2}\right)\left(1-\epsilon \right)}{\mu \left(\gamma_{1}+\mu \right)\left(\mu +\nu \right)\left(\mu +\xi \right)\left(\mu +\rho \right)\left(\left(1-\sigma_{2}\right)\lambda_{1}^{*}+\mu +\sigma_{2}\right)},\\ B^{*} & = & \frac{\pi \xi \epsilon }{{\left(\mu +\xi \right)}^{2}},\\ S_{m}^{*} & = & \frac{\pi \xi^{2}\epsilon }{\left(\lambda_{2}^{*}+\mu \right){\left(\mu +\xi \right)}^{2}},\\ E_{m}^{*} & = & \frac{\pi \lambda_{2}^{*}\xi^{2}\epsilon }{\left(\gamma_{2}+\mu \right)\left(\lambda_{2}^{*}+\mu \right){\left(\mu +\xi \right)}^{2}},\\ I_{m}^{*} & = & \frac{\pi \gamma_{2}\lambda_{2}^{*}\xi^{2}\epsilon }{\left(\gamma_{2}+\mu \right)\left(\kappa +\mu \right)\left(\lambda_{2}^{*}+\mu \right){\left(\mu +\xi \right)}^{2}},\\ R_{m}^{*} & = & \frac{\pi \gamma_{2}\kappa \lambda_{2}^{*}\xi^{2}\epsilon }{\mu \left(\gamma_{2}+\mu \right)\left(\kappa +\mu \right)\left(\lambda_{2}^{*}+\mu \right){\left(\mu +\xi \right)}^{2}}, \end{array}\right)$$

where, $\lambda_{2}^{*}=\frac{\pi \gamma_{1}\lambda_{1}\xi^{2}\left(1-\sigma_{1}\right)\left(1-\sigma_{2}\right)\left(1-\epsilon \right)\beta_{f\to m}}{\left(\gamma_{1}+\mu \right){\left(\mu +\xi \right)}^{2}\left(\left(1-\sigma_{2}\right)\lambda_{1}^{*}+\mu +\sigma_{2}\right)\left(\mu +\sigma_{1}+\left(1-\sigma_{1}\right)\xi \right)N_{f}}$ and$\lambda_{1}^{*}=\frac{\pi \gamma_{2}\lambda_{2}\xi^{2}\epsilon \beta_{m\to f}}{\left(\gamma_{2}+\mu \right)\left(\kappa +\mu \right)\left(\lambda_{2}+\mu \right){\left(\mu +\xi \right)}^{2}N_{m}}$. Substituting $\lambda_{2}^{*}$ into $\lambda_{1}^{*}$ and after some algebraic manipulations, we have a polynomial like:

(21) $$a_{1}a_{2}\mu \lambda_{1}^{*3}+a_{1}\left(a_{2}\mu^{2}+\pi \gamma_{2}\xi^{2}\left(1-\sigma_{2}\right)\epsilon \beta_{m\to f}\right)\lambda_{1}^{*2}+\left(a_{2}\sigma_{2}+\frac{a_{3}}{a_{2}a_{1}}\right)\lambda_{1}^{*}=0.$$

Let $z_{1}=a_{1}a_{2}\mu ,\;z_{2}=a_{1}\left(a_{2}\mu^{2}+\pi \gamma_{2}\xi^{2}\left(1-\sigma_{2}\right)\epsilon \beta_{m\to f}\right)\;\text{and}z_{3}=\left(a_{2}\sigma_{2}+\frac{a_{3}}{a_{2}a_{1}}\right)=\frac{\left(\mu +\kappa \right)\left(\mu +\gamma_{2}\right)\left(\mu +\nu \right)\left(\mu +\rho \right)\left(\mu +\gamma_{1}\right)\left[\left(1-ɛ\right)\xi^{2}+\left(\mu +\sigma_{2}\right)\sigma_{1}\right]}{\pi^{2}\gamma_{1}\xi^{2}\gamma_{2}\left(1-\sigma_{1}\right)\left(1-\sigma_{2}\right)\left(1-\epsilon \right)\epsilon \beta_{m\to f}\beta_{f\to m}}\left(1-R_{e}^{2}\right)$,whereby;

$$a_{1}=\left(\gamma_{1}+\mu \right){\left(\mu +\xi \right)}^{4}\left(\mu +\rho \right)\left(\mu +\left(1-\xi \right)\sigma_{1}+\xi \right)N_{f},$$

$$a_{2}=\left(\gamma_{2}+\mu \right)\left(\kappa +\mu \right){\left(\mu +\xi \right)}^{2}N_{m},$$

$$a_{3}=\pi^{2}\gamma_{1}\xi^{4}\gamma_{2}\left(1-\sigma_{1}\right)\left(1-\sigma_{2}\right)\left(1-\epsilon \right)\epsilon \beta_{m\to f}\beta_{f\to m}.$$

(22) $$z_{1}\lambda_{1}^{*3}+z_{2}\lambda_{1}^{*2}+z_{3}\lambda_{1}^{*}=0⟹\lambda_{1}^{*}\left(z_{1}\lambda_{1}^{*2}+z_{2}\lambda_{1}^{*}+z_{3}\right)=0.$$

For $\lambda_{1}^{*}=0$, corresponds to DFE, whereas Eqn. (22) can also be written in the form:

(23) $$\lambda_{1}^{*}=\frac{-z_{2}\pm \sqrt{z_{2}^{2}-4z_{1}z_{3}}}{2z_{1}},$$

which satisfies EE. The value of $z_{1}$ is strictly positive. Depending on the signs of $z_{2}$ and $z_{3}$, there are three cases to consider as having positive real root of $\lambda_{1}^{*}$ as follows:**Case 1:** If $z_{2}<0$ then system (1) has a stable endemic equilibrium point when $z_{3}<0$.**Case 2:** Exactly one endemic equilibrium point.Suppose $z_{2}<0$ and $z_{3}=0$ or $z_{2}^{2}-4z_{1}z_{3}=0$. In other words, the polynomial $z_{1}\lambda_{1}^{*2}+z_{2}\lambda_{1}^{*}+z_{3}=0$ has just one positive root and hence the system (1) has unique endemic equilibrium point.**Case 3:** Two endemic equilibria.If $z_{2}<0$, $z_{3}>0$ and $z_{2}^{2}-4z_{1}z_{3}>0$, then the polynomial $z_{1}\lambda_{1}^{*2}+z_{2}\lambda_{1}^{*}+z_{3}=0$ has two positive real roots. In other words, the system (1) has two endemic equilibria and hence there is a possibility of backward bifurcation. These three cases are summarised here under

**Fig. 7:**
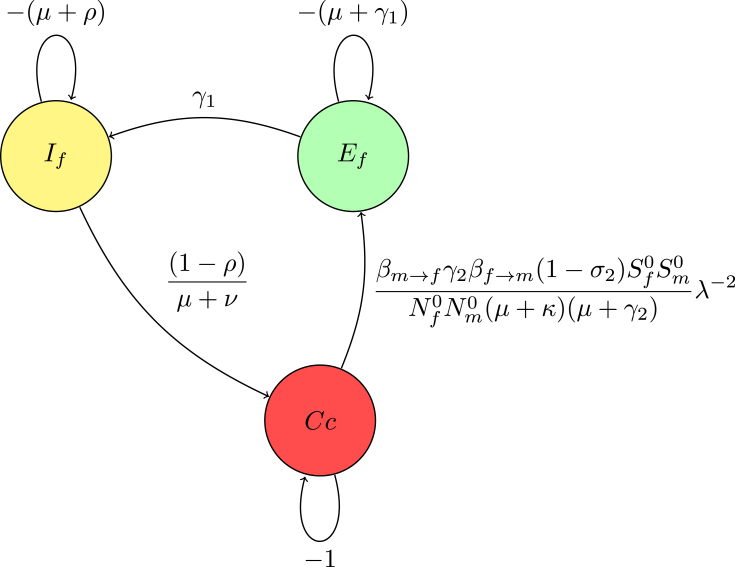
(**C**) Creating a trivial node at node Cc.

**Fig. 8:**
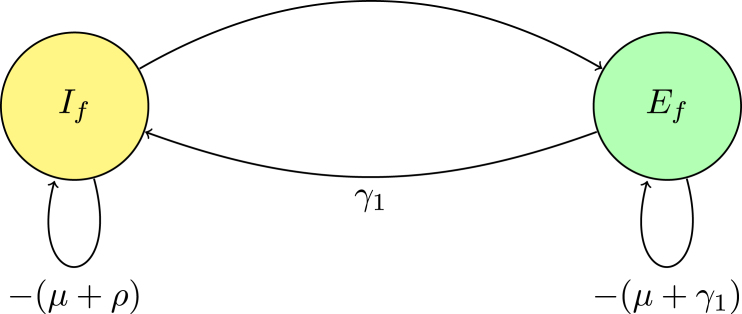
(D*) Digraph Representation without node Cc.

**Fig. 9:**
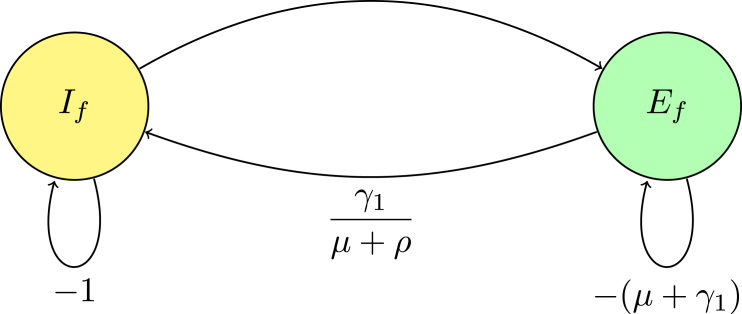
(**D**) Creating a trivial node at node $I_{f}$.

**Fig. 10:**
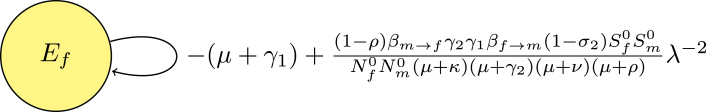
(**E**) Digraph representation without node $I_{f}$.

**Fig. 11:**
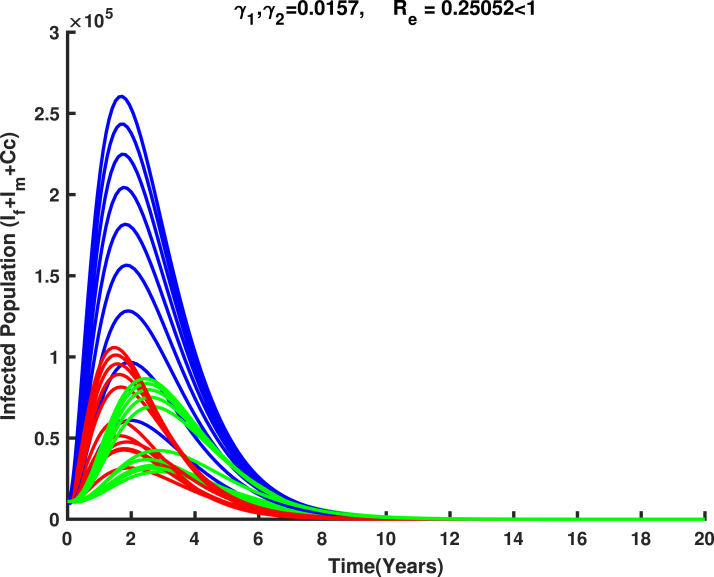
Stability of disease free equilibrium in Ω for $R_{e}<1$.

(i) If $z_{3}<0$, $R_{e}>1$, then the system has a unique endemic equilibrium.
(ii) If $z_{2}<0$ and $z_{3}=0$ or $z_{2}^{2}-4z_{1}z_{3}=0$, then the system has exactly one endemic equilibrium.
(iii) If $z_{2}<0$, $z_{3}>0$ and $z_{2}^{2}-4z_{1}z_{3}>0$, then the system has exactly two endemic equilibria.
(iv) Otherwise there are no endemic equilibria, i.e. when $z_{1}z_{3}>0$ and $z_{2}>0$.

### Global stability at the endemic equilibrium point

It is demonstrated that the endemic equilibrium point $E^{*}$ is globally asymptotically stable when $R_{e}>1$. The endemic equilibrium point $E^{*}=\left(C^{*},V^{*},S^{*},E^{*},I^{*},Cc^{*},R^{*}\right)$ lies within the interior region $\Omega^{0}$ where all population states remain biologically and epidemiologically feasible.

In accordance with the uniform persistence theorem [48] and the result stated in Proposition 3.3 of [49], the instability of the disease-free equilibrium $E_{0}$ guarantees the uniform persistence of system (1) in $\Omega^{0}$. Furthermore, the solutions of system (1) are uniformly bounded in the feasible region $\Omega^{0}$, which follows from (5) of the solutions in $\Omega^{0}$. The uniform persistence of system (1) and the boundedness of solutions in $\Omega^{0}$ confirm the existence of endemic equilibria $E^{*}$ in $\Omega^{0}$ [50]. Biologically, when $R_{e}>1$, the disease will persist in the population at a unique endemic state. A lemma provided in [51] establishes the global stability of the endemic equilibrium point.

Lemma 1 *Consider that the matrix* $H_{5\times 5}$ *is irreducible. Then, the linear system*

(24) Hv=0

*has the following properties:*

*1.* *The solution space of the linear system* (24) *in* $\Omega^{0}$ *has a dimension one.*
*2.* *The basis of the solution space is* $v=\left(v_{1},v_{2},\ldots ,v_{5}\right)=\left(v_{11},v_{22},\ldots ,v_{55}\right),$ *where* $v_{kk}$ *is the* k *th diagonal elements of matrix* H *, with* 1≤k≤5 *.*

Theorem 5 *Assume that the graph associated with the matrix* H *is strongly connected and that* $R_{e}>1$ *. Then, the endemic equilibrium of system* (1) *is globally asymptotically stable in* $\Omega^{0}$ *.*

Proof To establish the global stability of the endemic equilibrium $E^{*}$, the irreducibility of $M_{0}$ implies that H is irreducible, and $v_{k}>0$ for k=1,…,n. A nonnegative function, which considers the classes where infection may arise, along with the susceptible class in the model system (1), is introduced based on the works [52], [53]. A Goh-Volterra Lyapunov function $P:\Omega^{0}\to R_{+}$ is defined such that:

(25) $$P=\overset{n}{\underset{k=1}{\sum }}v_{k}\left(S-S^{*}-S^{*}\ln\frac{S}{S^{*}}+E-E^{*}-E^{*}\ln\frac{E}{E^{*}}+I-I^{*}-I^{*}\ln\frac{I}{I^{*}}+Cc-Cc^{*}-Cc^{*}\ln\frac{Cc}{Cc^{*}}\right)$$

with a condition thatΥ(y)=1−y+lny≤0:Υ(y)=0⟺y=1 where (S,E,I,Cc)∈y

Differentiating P along the solutions of system (1), it is obtained that

$$\frac{dP}{dt}=\overset{n}{\underset{k=1}{\sum }}v_{k}\left(\left(1-\frac{S^{*}}{S}\right)\frac{dS}{dt}+\left(1-\frac{E^{*}}{E}\right)\frac{dE}{dt}+\left(1-\frac{I^{*}}{I}\right)\frac{dI}{dt}+\left(1-\frac{Cc^{*}}{Cc}\right)\frac{dCc}{dt}\right)=v_{1}\left(1-\frac{S_{f}^{*}}{S_{f}}\right)\left(\xi \left(1-\sigma_{1}\right)G-\mu S_{f}-\sigma_{2}S_{f}-\left(1-\sigma_{2}\right)\lambda_{1}S_{f}\right)+v_{2}\left(1-\frac{S_{m}^{*}}{S_{m}}\right)\left(\xi B-\mu S_{m}-\lambda_{2}S_{m}\right)+\;v_{3}\left(1-\frac{E_{f}^{*}}{E_{f}}\right)\left(\left(1-\sigma_{2}\right)\lambda_{1}S_{f}-\left(\mu +\gamma_{1}\right)E_{f}\right)+v_{4}\left(1-\frac{E_{m}^{*}}{E_{m}}\right)\left(\lambda_{2}S_{m}-\left(\mu +\gamma_{2}\right)E_{m}\right)+\;v_{5}\left(1-\frac{I_{f}^{*}}{I_{f}}\right)\left(\gamma_{1}E_{f}-\left(\mu +\rho \right)I_{f}\right)+\left(1-\frac{I_{m}^{*}}{I_{m}}\right)\left(\gamma_{2}E_{m}-\left(\mu +\kappa \right)I_{m}\right)+\;v_{6}\left(1-\frac{Cc^{*}}{Cc}\right)\left(\left(1-\rho \right)I_{f}-\left(\mu +\nu \right)Cc\right)=v_{1}\left(1-\frac{S_{f}^{*}}{S_{f}}\right)\left(\mu S_{f}^{*}+\sigma_{2}S_{f}^{*}+\left(1-\sigma_{2}\right)\lambda_{1}^{*}S_{f}^{*}-\mu S_{f}-\sigma_{2}S_{f}-\left(1-\sigma_{2}\right)\lambda_{1}S_{f}\right)+\;v_{2}\left(1-\frac{S_{m}^{*}}{S_{m}}\right)\left(\mu S_{m}^{*}+\lambda_{2}^{*}S_{m}^{*}-\mu S_{m}-\lambda_{2}S_{m}\right)+\;v_{3}\left(1-\frac{E_{f}^{*}}{E_{f}}\right)\left(\left(\mu +\gamma_{1}\right)E_{f}^{*}-\left(\mu +\gamma_{1}\right)E_{f}\right)+v_{4}\left(1-\frac{E_{m}^{*}}{E_{m}}\right)\left(\left(\mu +\gamma_{2}\right)E_{m}^{*}-\left(\mu +\gamma_{2}\right)E_{m}\right)+\;v_{5}\left(1-\frac{I_{f}^{*}}{I_{f}}\right)\left(\gamma_{1}E_{f}-\left(\mu +\rho \right)I_{f}\right)+\left(1-\frac{I_{m}^{*}}{I_{m}}\right)\left(\gamma_{2}E_{m}-\left(\mu +\kappa \right)I_{m}\right)+\;v_{6}\left(1-\frac{Cc^{*}}{Cc}\right)\left(\left(\mu +\nu \right)Cc^{*}-\left(\mu +\nu \right)Cc\right)=v_{1}\left(\left(1-\sigma_{2}\right)\left(\lambda_{1}^{*}\left(1-\frac{S_{f}^{*}}{S_{f}}\right)+\lambda_{1}\left(1-\frac{S_{f}}{S_{f}^{*}}\right)\right)+\left(\mu +\sigma_{2}\right)\left(2-\frac{S_{f}^{*}}{S_{f}}-\frac{S_{f}}{S_{f}^{*}}\right)\right)S_{f}^{*}+\;v_{2}\left(\lambda_{2}^{*}\left(1-\frac{S_{m}^{*}}{S_{m}}\right)+\lambda_{2}\left(1-\frac{S_{m}}{S_{m}^{*}}\right)+\mu \left(2-\frac{S_{m}^{*}}{S_{m}}-\frac{S_{m}}{S_{m}^{*}}\right)\right)S_{m}^{*}+\;v_{3}\left(\mu +\gamma_{1}\right)\left(2-\frac{E_{f}^{*}}{E_{f}}-\frac{E_{f}}{E_{f}^{*}}\right)E_{f}^{*}+v_{4}\left(\mu +\gamma_{2}\right)\left(2-\frac{E_{m}^{*}}{E_{m}}-\frac{E_{m}}{E_{m}^{*}}\right)E_{m}^{*}+\;v_{5}\left(\mu +\rho \right)\left(2-\frac{I_{f}^{*}}{I_{f}}-\frac{I_{f}}{I_{f}^{*}}\right)I_{f}^{*}+v_{6}\left(\mu +\kappa \right)\left(2-\frac{I_{m}^{*}}{I_{m}}-\frac{I_{m}}{I_{m}^{*}}\right)I_{m}^{*}+v_{7}\left(\mu +\nu \right)\left(2-\frac{Cc^{*}}{Cc}-\frac{Cc}{Cc^{*}}\right)Cc^{*}$$

It follows from (5) that the expressions $\left(2-\frac{S_{f}^{*}}{S_{f}}-\frac{S_{f}}{S_{f}^{*}}\right)\leq 0,\;\left(2-\frac{S_{m}^{*}}{S_{m}}-\frac{S_{m}}{S_{m}^{*}}\right)\leq 0,$ $\;\left(2-\frac{E_{f}^{*}}{E_{f}}-\frac{E_{f}}{E_{f}^{*}}\right)\leq 0,\;$ $\left(2-\frac{E_{m}^{*}}{E_{m}}-\frac{E_{m}}{E_{m}^{*}}\right)\leq 0,\;\left(2-\frac{I_{f}^{*}}{I_{f}}-\frac{I_{f}}{I_{f}^{*}}\right)\leq 0,\;\left(2-\frac{I_{m}^{*}}{I_{m}}-\frac{I_{m}}{I_{m}^{*}}\right)\leq 0,\;\text{and}$ $\;\left(2-\frac{Cc^{*}}{Cc}-\frac{Cc}{Cc^{*}}\right)\leq 0.$

$$\frac{dP}{dt}\;\leq \;v_{1}\left(1-\sigma_{2}\right)\left(\lambda_{1}^{*}\left(1-\frac{S_{f}^{*}}{S_{f}}\right)+\lambda_{1}\left(1-\frac{S_{f}}{S_{f}^{*}}\right)\right)S_{f}^{*}+v_{2}\left(\lambda_{2}^{*}\left(1-\frac{S_{m}^{*}}{S_{m}}\right)+\lambda_{2}\left(1-\frac{S_{m}}{S_{m}^{*}}\right)\right)S_{m}^{*}$$

By using the inequality 1−y≤−lny, it follows that

$$\frac{dP}{dt}\;\leq \;-v_{1}\left(1-\sigma_{2}\right)\left(\lambda_{1}^{*}\ln\frac{S_{f}^{*}}{S_{f}}+\lambda_{1}\ln\frac{S_{f}}{S_{f}^{*}}\right)S_{f}^{*}-v_{2}\left(\lambda_{2}^{*}\ln\frac{S_{m}^{*}}{S_{m}}+\lambda_{2}\ln\frac{S_{m}}{S_{m}^{*}}\right)S_{m}^{*}$$

≤0

The equality holds if and only if $S=S^{*}$, $E=E^{*}$, $I=I^{*}$, and $Cc=Cc^{*}$. Consequently, P serves as a Lyapunov function on $\Omega^{0}$, and $E^{*}$ is the largest invariant set for which $\frac{dP}{dt}=0$. By LaSalle’s invariance principle [47], $E^{*}$ is globally stable in $\Omega^{0}$.

**Fig. 12:**
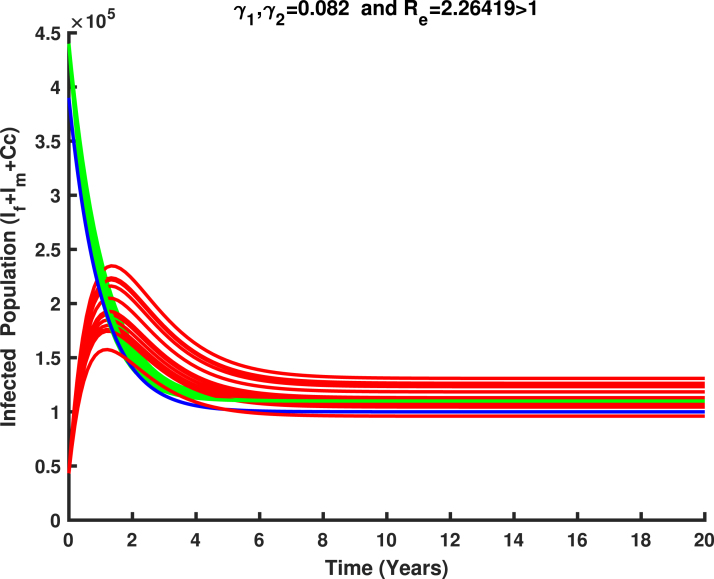
Stability of endemic equilibrium in $\Omega^{0}$ for $R_{e}>1$.

Epidemiologically, based on the above analysis, we can confidently conclude that HPV will spread within the population if the value of $R_{e}$ exceeds unity. The uncontrolled spread of HPV increases the risk of cervical cancer, leading to significant mortality. At this point, the disease cannot be eradicated and the burden of cervical cancer and its associated deaths will continue to rise if no measures are taken. Fig. 12 demonstrates the validation of the global stability analysis for the endemic equilibrium point. □

### Parameter estimation using markov chain monte carlo

The parameters described in model system (1) were estimated using synthetic data through a least squares fitting method, where variables of interest are $V_{1},V_{2},E_{f},I_{f},R_{f}$ and Cc as defined in Eq. (1). These synthetic data points were generated based on parameters as in Table 1 from existing literature, allowing for the simulation of the model system (1). The estimation process involves minimizing the residual sum of squares (RSS), which is given by:

(26) $$\text{RSS}=\overset{n}{\underset{i=1}{\sum }}{\left(Y_{i}-f\left(Y_{i},\theta \right)\right)}^{2},$$

where, $Y_{i}$ represent the synthetic data points, $f\left(Y_{i},\theta \right)$ the model function with parameter θ, and n is the total number of synthetic data points.

The objective is to find the parameter values $\theta =\left(\sigma_{1},\sigma_{2},\gamma_{1},\nu ,\rho \right)$ that minimize the RSS, based on the synthetic data, and estimate model parameters that are consistent with values from the literature. From [17], [38], it is assumed that there $E_{f_{0}}=100\;000$, $S_{f_{0}}=5\;365\;499$, $V_{1_{0}}=600\;000$, $V_{2_{0}}=0$, $I_{f_{0}}=0$, $R_{f_{0}}=0$, and $Cc_{0}=10868$.

In accordance with condition (7), approximately Tanzania has a population of 66617606, with an estimated life expectancy of an individual 66.8, we can now estimate natural death rate (μ) and recruitment rate (π).

(27) $$\mu =\frac{1}{\text{Life expectancy}}⟹\mu =\frac{1}{66.8}\approx 0.015.$$

$$N\left(t\right)\leq \frac{\pi }{\mu }⟹\pi =66617606\times 0.015\approx 999264$$

As from [54], [55] and Table 1, initial parameter values are constrained such that $\left(\sigma_{1},\sigma_{2},\gamma_{1},\nu ,\rho \right)\in \left[0,1\right]$. Parameter estimates are listed in Table 3. The standard deviation (STD) of the parameter estimates were calculated in the standard way, by the diagonal of the approximative covariance matrix via the Jacobian matrix.

These estimates were used as the starting point for a more comprehensive MCMC study of the parameter identifiability. The Metropolis algorithm, as discussed in [54], [55], [56], was applied. The Markov Chain Monte Carlo (MCMC) method involved sampling from probability distributions by constructing Markov chains that converge to the posterior distribution. This allowed simulating the entire joint posterior distribution of the unknown quantities and obtaining simulation-based estimates of the posterior parameters of interest. The MCMC chain also reveals the unidentifiability of model parameters, which arises due to parameter correlation and model nonlinearity [57]. The MCMC process yields a matrix of size numberofsimulations×numberofparametersdistributed. Herein, we have 5 parameters and 5000 simulations. This large number of simulations allows for the generation of long chains, enabling the assessment of inaccuracies in the model and the production of better graphical representations. The posterior mean and posterior standard deviation for the estimated parameters are reported in Table 4, along with their corresponding nominal 95% confidence intervals.

**Table 3:** Estimated HPV model parameters using synthetic data by least square method.

Parameter	Description	Initial values	Estimates	STD
$\sigma_{1}$	Vaccination rate for girls	0.016	0.03356	0.0012551
$\sigma_{2}$	Vaccination rate female	0.87	0.0056	0.00017645
ρ	Recovered female	0.15	0.00015	$5.0011\times 10^{5}$
ν	The disease induced rate	0.01	0.0345	0.000167
$\gamma_{1}$	Progression rate	0.0157	0.082	0.011303

**Fig. 13:**
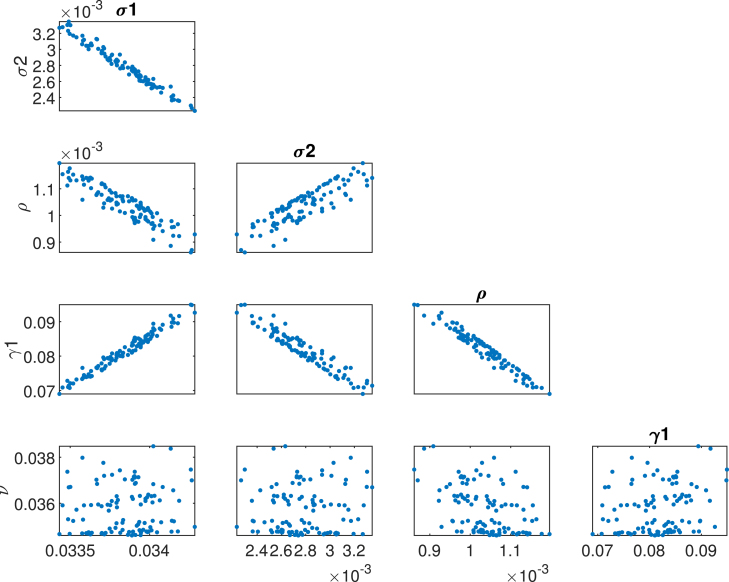
Pairwise scatter plots for the unknown parameters $\;\sigma_{1},\;\sigma_{2},\;\rho ,\;\nu \;\text{and}\;\gamma_{1}$.

The Adaptive Metropolis algorithm (AM) [55] was employed in this study. The initial proposal distribution was defined by the covariance matrix obtained through the least squares fit method. Through the MCMC figures, it is possible to extract information about the correlation, uncertainty, parameter identifiability, and convergence of the Markov chain to the target distribution. Fig. 13 presents the time-series plot for each parameter, showing that parameters $\left(\sigma_{1}\text{and}\gamma_{1}\right)$ and $\left(\sigma_{2}\text{and}\rho \right)$ are positively correlated. This indicates that vaccinating females aged above 14 years has a more significant impact on recovery rates compared to vaccinating girls aged 9–14 years. The increase in vaccination rates for older females corresponds to an increase in the recovery rate. Conversely, a negative correlation is observed with mass vaccination of girls aged 9–14 years. An increase in vaccination coverage for this younger age group leads to a long-term reduction in vaccination rates, as most females are vaccinated at a younger age. Consequently, the recovery rate decreases because fewer individuals are observed in the exposed or infected classes over time.

**Table 4:** Posterior mean and standard deviation of the estimated parameters.

Parameter	Posterior mean	Posterior STD
$\sigma_{1}$	0.0356(0.0322−0.0342)	0.0012551
$\sigma_{2}$	0.0056(0.0026−0.00649)	0.0001764
ρ	0.00015(0.0008−0.00119)	$5.0011\times 10^{5}$
ν	0.0345(0.0344−0.0375)	0.000167
$\gamma_{1}$	0.082(0.0675−0.0979)	0.011303

**Fig. 14:**
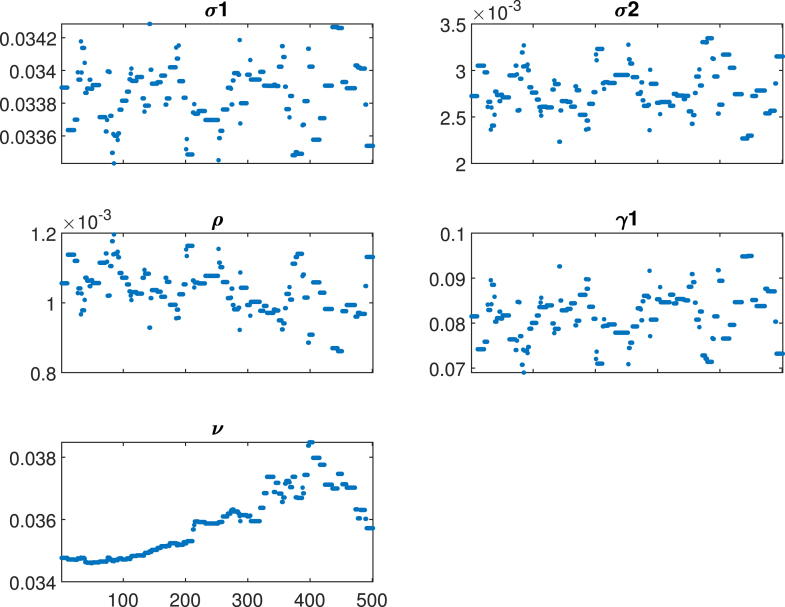
Trace plots of estimated unknown ($\sigma_{1},\;\sigma_{2},\;\rho ,\;\nu \;\text{and}\;\gamma_{1}$) parameters using MCMC.

The identifiability of key parameters in the HPV transmission and cervical cancer model was evaluated through trace plots, posterior distributions, and pairwise parameter correlations obtained from the MCMC analysis. As in Fig. 14, the parameter $\gamma_{1}$ showed good identifiability, with posterior distributions indicating that the data provides sufficient information on the rate at which exposed individuals progress to infection. This reflects the importance of early detection and monitoring of HPV exposure in understanding transmission dynamics. However, some correlation with other parameters, particularly ν (mortality rate), suggests potential challenges in distinguishing progression dynamics from downstream effects such as cancer development. The identifiability of $\sigma_{1}$ was moderate. The posterior distribution was relatively broad, suggesting some uncertainty in estimating the impact of vaccinating girls in this age group. This may result from the overlap in effects between $\sigma_{1}$ and $\sigma_{2}$ (vaccination of females aged above 14 years). In biological terms, this implies that the long-term benefits of vaccinating younger girls are intertwined with vaccination coverage in older females, emphasizing the need to isolate their independent contributions to reducing HPV prevalence. The parameter $\sigma_{2}$ was strongly correlated with ρ (recovery rate), indicating a synergistic relationship where increasing vaccination rates in older females corresponds to higher recovery rates. This correlation highlights the role of vaccination in reducing the active infection pool and facilitating natural or treatment-driven recovery. However, the identifiability of $\sigma_{2}$ was somewhat compromised by its overlap with $\sigma_{1}$, pointing to challenges in separating age-specific vaccination impacts. The recovery rate ρ was well-identified, with narrow posterior distributions indicating strong data support. This parameter’s correlation with vaccination rates $\sigma_{2}$ underscores the impact of vaccination in facilitating recovery, either by preventing reinfection or enhancing the immune response. Biologically, this suggests that recovery dynamics are a critical component of HPV control and should be monitored alongside vaccination coverage. The parameter ν showed poor identifiability, with broad posterior distributions and weak convergence. This reflects the complexity of linking HPV dynamics to cervical cancer mortality, particularly in settings where data on advanced stage cancer and treatment outcomes is sparse.

### Herd immunity

Herd immunity refers to the indirect or direct protection from an infectious disease that occurs when a significant portion of a population becomes immune, either through natural infection or artificial inoculation with vaccine. This reduces the number of susceptible individuals, thereby lowering the overall risk of infection. With fewer people capable of contracting and transmitting the disease, the chain of transmission is disrupted, effectively controlling the disease’s spread within the community. However, understanding the reproductive number of an infection is useful in the concept of herd immunity [58], [59], [60].

**Fig. 15:**
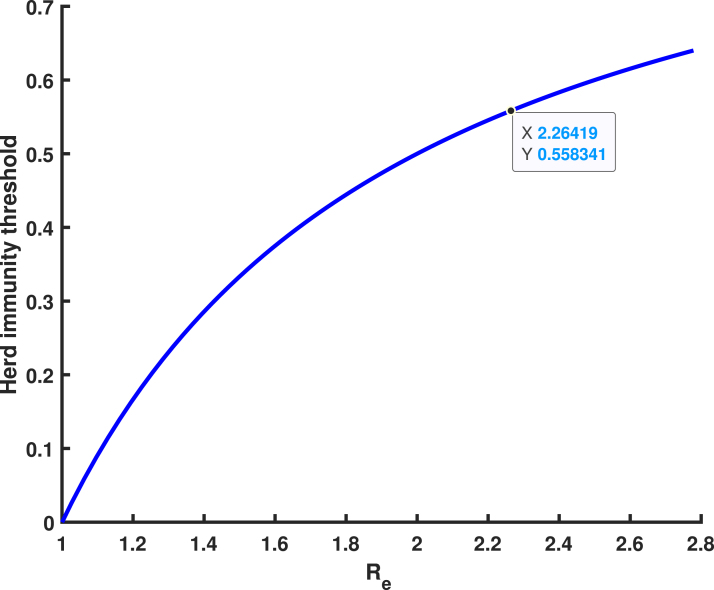
Reproduction number against herd immunity.

Mathematically, the herd immunity threshold represents the proportion of the population that must be immune to prevent the disease from spreading. When herd immunity is achieved through effective vaccination or immunization, each case of the disease results in only one new case ($R_{e}=1$), leading to a stable infection rate within the population. If the immunity level exceeds the herd immunity threshold, $R_{e}$ falls below 1, causing a decline in infection cases. Thus, herd immunity is crucial for controlling HPV transmission and serves as a foundation for planning vaccination and eradication programs. The herd immunity threshold $R_{h}\left(t\right)$ at any time t is expressed as: $R_{h}\left(t\right)=\left(1-\frac{1}{R_{e}}\right)$. When $R_{e}=2.26419$, Fig. 15 illustrates a geometric increase in infections over time if no individuals are immune. This implies that approximately 55.83% of the population must be immune to achieve herd immunity and prevent the spread of HPV.

Tanzania targets vaccinating girls aged 9–14 years, a crucial group as they are less likely to have been exposed to HPV. To achieve herd immunity, 55.83% vaccination efforts should be extended to women aged 15–49 as this group is at higher risk due to sexual debut, which often occurs in the reproductive age range. This makes the 15–49 age group particularly vulnerable to HPV transmission.

**Fig. 16:**
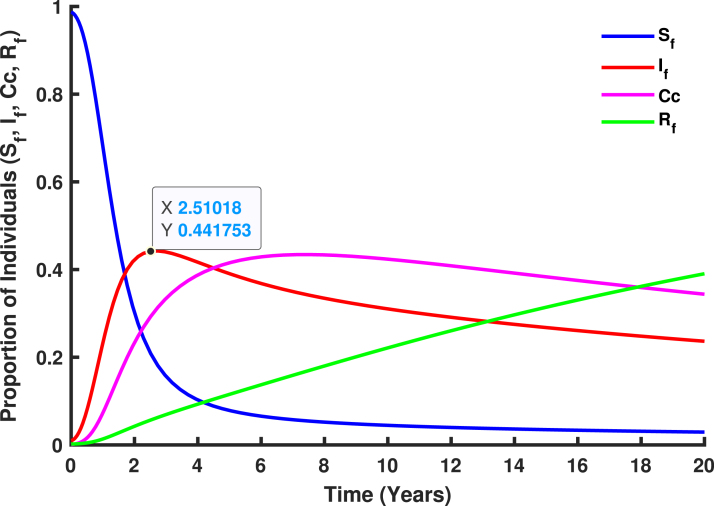
An homogeneous proportion. (For interpretation of the references to color in this figure legend, the reader is referred to the web version of this article.)

**Fig. 17:**
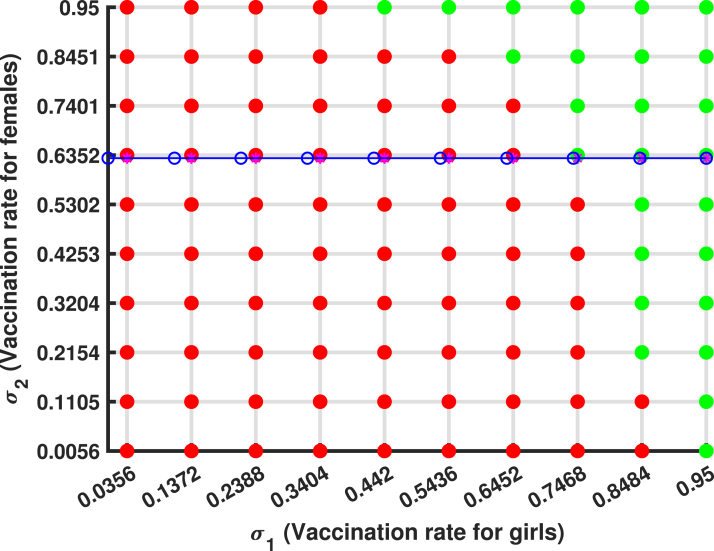
Optimal vaccination rate.

Consider Fig. 16 with $R_{e}=2.26419$, illustrating a homogeneous population mixing scenario where individuals of reproductive age face a significant risk of developing cervical cancer. When a single infected individual is introduced, the proportion of infected individuals (depicted by the red line) rises rapidly, peaking at the herd immunity threshold of 0.4417 (44.17%). Beyond this threshold, each newly infected individual transmits the infection to fewer than one susceptible individual due to an increase in vaccination rates, optimized to $\left(\sigma_{1},\sigma_{2}\right)\to \left(0.7468,0.6352\right)$, as shown in Fig. 17. At this stage, a sufficient proportion of the population has developed immunity, reducing $R_{e}$ to approximately 1. As a result, the required number of vaccinations decreases, and the number of individuals to be vaccinated diminishes ($R_{h}\left(t\right)\to 0$), effectively stopping further HPV transmission within the population.

Expanding HPV vaccination to include unvaccinated girls and female is essential to reduce the 44.17% of the population still at risk. Herd immunity decreases HPV prevalence, minimizing exposure risks for vulnerable individuals and indirectly protecting the unvaccinated by limiting virus circulation and community transmission. Female aged 15–49 years, the reproductive age group is at higher risk due to sexual activity, as well as girls who missed vaccine at the age 9–14 and individuals in underserved areas, face the greatest vulnerability. Vaccination not only reduces HPV infections linked to cervical cancer but also significantly lowers cervical cancer risk. Achieving herd immunity by vaccinating at least 55.83% of the population safeguards unvaccinated individuals and curbs HPV transmission, ultimately reducing the burden of cervical cancer in Tanzania, saving lives, and lowering healthcare costs.

Fig. 11, Fig. 12, Fig. 13, Fig. 14, Fig. 15, Fig. 16, Fig. 17 was influenced by Markov Chain Monte Carlo (MCMC) methods were used for parameter estimation to calibrate the model and reflect realistic HPV dynamics and vaccination impacts. This approach accounts for uncertainties in parameter values and ensures that the model outputs align with observed HPV transmission patterns and vaccination outcomes. Robust estimates for parameters such as vaccination rates, progression rate, recovery rate, and mortality rate were determined. These estimates shaped the trajectories of the compartments by capturing reductions in susceptibility, infection prevalence, and cervical cancer cases. Incorporating uncertainty in parameter estimation ensures the variability in population behavior and intervention effectiveness, resulting in credible and actionable outputs for public health planning.

### Numerical simulation of HPV transmission dynamics and the impact of vaccination

**Fig. 18:**
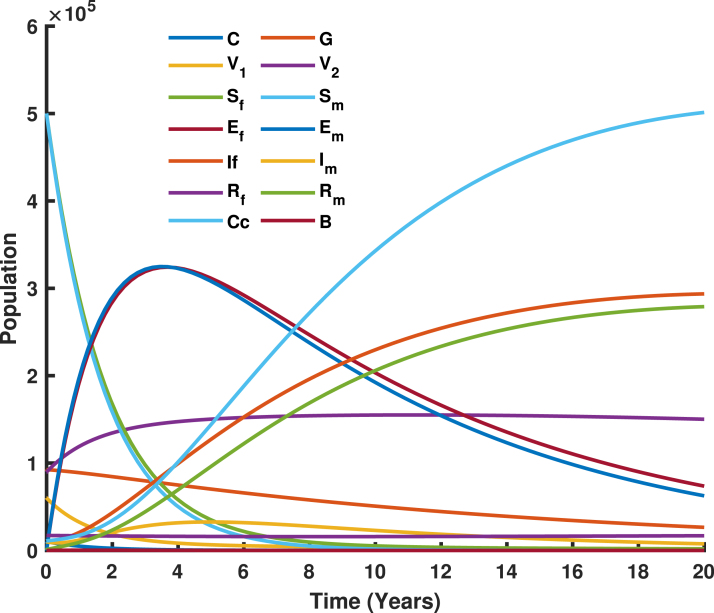
Population dynamics of females and males, including age-specific distributions of girls and boys, and their impact on HPV transmission and vaccination outcomes.

Fig. 18 illustrate the transmission of human papillomavirus (HPV) and its progression to cervical cancer through various population compartments. Fig. 1 highlights the transitions between susceptible females ($S_{f}$), infected females ($I_{f}$), recovered females ($R_{f}$), and those who progress to cervical cancer ($C_{c}$). This framework captures the key elements influencing HPV prevalence and the burden of cervical cancer in Tanzania. HPV transmission involves both female and male populations, where males serve as a reservoir for the virus and play a critical role in transmitting HPV to susceptible females ($S_{f}$). Infected males ($I_{m}$) contribute to the persistence and spread of the virus within the population, emphasizing the need to consider their role in the dynamics. The interplay between infected males and females sustains the infection cycle, particularly in the absence of male-targeted interventions.

**Fig. 19:**
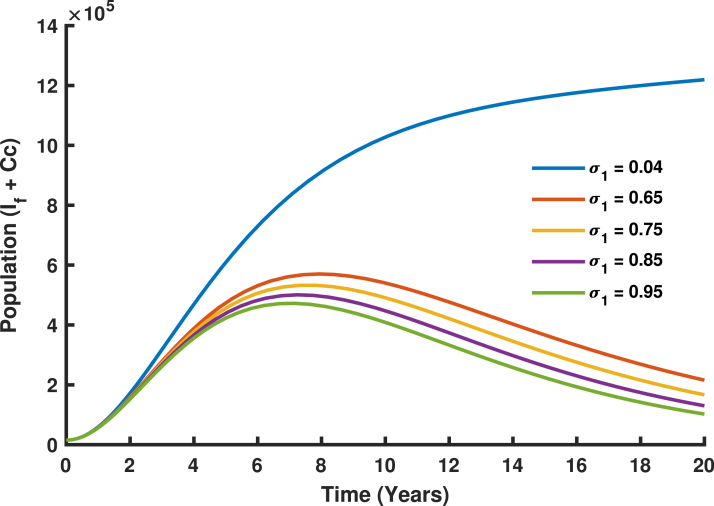
Varying vaccination sigma ($\sigma_{1}$).

The vaccination rate for girls aged 9–14 years $\sigma_{1}$ has a less significant impact on reducing HPV prevalence and cervical cancer cases. This underscores the necessity of incorporating other age group so that both girls and female are immunized before potential exposure to HPV, the primary cause of cervical cancer. However, implementing only vaccine in girls does not provide herd immunity to those who missed the vaccine. Analysis of the model, as illustrated in Fig. 19, shows that higher $\sigma_{1}$ values (e.g., 0.85 or 0.95) result in only a slight reduction in the population of infectious females ($I_{f}$) and cervical cancer cases (Cc). The gradual decline in Fig. 19 demonstrates that the $\left(I_{f}+Cc\right)$ population decreases slowly as $\sigma_{1}$ increases, emphasizing the critical need to target the older age group. While early vaccination creates a robust protective effect across the population over time, it is crucial to prioritize follow-up for those who missed the vaccine, as they remain at significant risk.

**Fig. 20:**
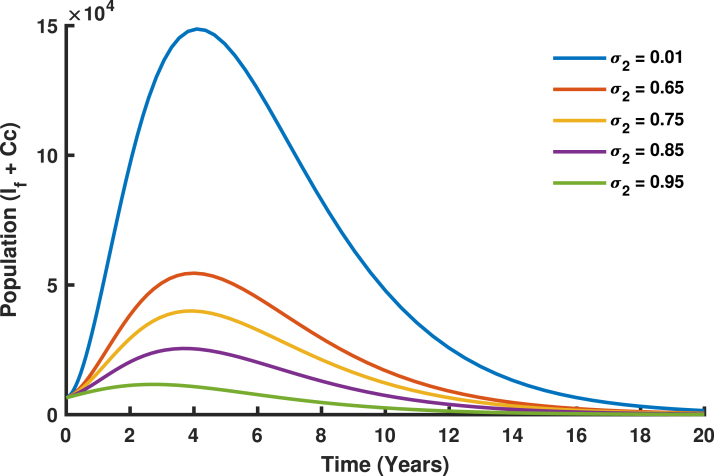
Varying vaccination rate ($\sigma_{2}$).

The vaccination rate for females above 14 years $\sigma_{2}$ also contributes to reducing HPV prevalence, with its impact being more pronounced than that of $\sigma_{1}$. Fig. 20 demonstrates that while higher $\sigma_{2}$ values (e.g., 0.85 and 0.95) lead to a reduction in cases, the decline is significantly more dramatic compared to $\sigma_{1}$. In Fig. 20, the $\left(I_{f}+C_{c}\right)$ population shows a more pronounced decrease over time as $\sigma_{2}$ values increase. This highlights the importance of vaccinating older females before exposure to HPV, as prior exposure reduces the vaccine’s preventive efficacy. Nevertheless, increasing $\sigma_{2}$ values plays a crucial role in lowering HPV prevalence, indicating that catch-up vaccination programs provide substantial benefits by reducing transmission and offering herd immunity to protect unvaccinated individuals.

**Fig. 21:**
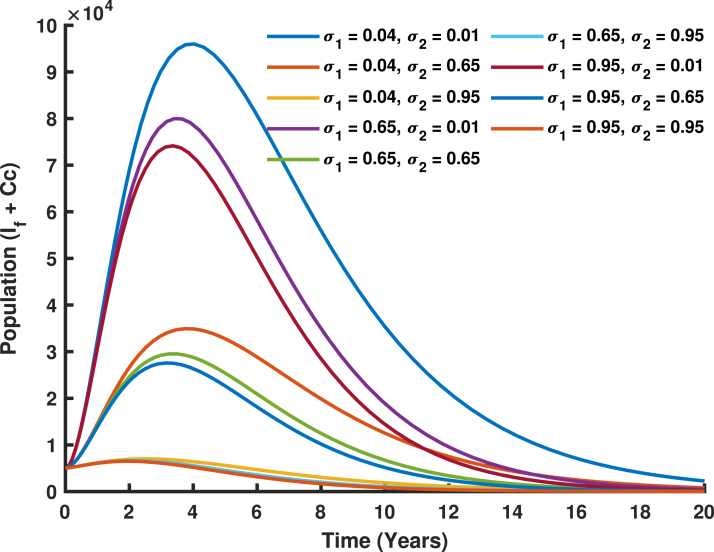
Varying vaccination rates ($\sigma_{1}$ and $\sigma_{2}$).

The combined use of vaccination rates $\sigma_{1}$, targeting girls aged 9–14 years, and $\sigma_{2}$, targeting females above 14 years, is a comprehensive and strategic intervention aimed at reducing human papillomavirus (HPV) transmission and cervical cancer in Tanzania. As illustrated in Fig. 21, $\sigma_{1}$ establishes early immunity by vaccinating girls before their sexual debut, which not only directly protects them but also limits the reservoir of susceptible individuals entering the sexually active population, thereby promoting herd immunity. Meanwhile, $\sigma_{2}$ acts as a vital catch-up strategy to protect older females who might have missed earlier vaccination opportunities or been exposed to HPV but are not yet infected, significantly reducing the progression of infections to cervical cancer. Together, these vaccination strategies create a layered defense, with $\sigma_{1}$ reducing the primary transmission of HPV and $\sigma_{2}$ targeting at risk populations, ensuring broader population level protection. The synergistic effect of combining these strategies is evident in the sharp decline in the populations of infected females and the incidence of cervical cancer over a 20-year period. This approach directly addresses Tanzania’s high HPV burden and cervical cancer mortality rates, which are exacerbated by limited access to screening and treatment services. By targeting distinct yet complementary age groups, the combined vaccination strategy aligns with global elimination goals, reducing the overall burden on Tanzania’s healthcare system and significantly improving the health and well being of female and girls.

### Discussion

The model’s findings emphasize the crucial role of the recovery rate ρ in reducing HPV prevalence and promoting recovery, suggesting that follow-up therapies after vaccination are essential for optimizing recovery outcomes. Additionally, the interaction between vaccination rates for younger girls $\sigma_{1}$ and older females $\sigma_{2}$ highlights the importance of a sequential vaccination strategy: vaccinating younger girls reduces the susceptible pool, while increasing coverage among older females boosts recovery rates, maximizing public health benefits. The analysis also suggests that tailoring vaccination strategies to high-prevalence subpopulations could improve effectiveness, and enhancing early detection through the progression rate $\gamma_{1}$, alongside addressing data gaps on the mortality rate ν, could further strengthen intervention strategies. These findings support the development of more targeted public health policies and precision medicine approaches, especially in resource-limited settings, by addressing key data gaps and advancing HPV control efforts.

Although the robustness of the proposed HPV transmission model needs validation using real-world data, including vaccination coverage and cervical cancer incidence in Tanzania, it indicated that maintaining vaccination rates beyond $\sigma_{1}=0.7468$ and $\sigma_{2}=0.6352$ can effectively keep HPV under control. The model was calibrated with synthetic data on vaccination rates, based on 79% of girls who received the first dose and 60% who received the second by 2023, alongside cervical cancer incidence (10,868 cases) and mortality data (6832 deaths). Comparing the model’s predictions with these data helped evaluate its accuracy, especially concerning infection reduction and herd immunity thresholds.

While we acknowledge inherent data uncertainties, such as insufficient coverage and inconsistent records, we recognize the potential biases these gaps may introduce. Although the model focuses on female vaccination, incorporating male vaccination into HPV transmission dynamics would provide a more comprehensive understanding. For simplicity, assumptions such as 100% vaccine efficacy and lifelong immunity were made. However, addressing factors like varying vaccine efficacy, waning immunity, and breakthrough infections will enhance model accuracy, as few vaccines achieve such high efficacy. Additionally, addressing logistical challenges such as rural vaccine distribution and conducting cost effectiveness analyses alongside cervical screening programs are crucial for developing robust strategies.

Community based education programs, targeted outreach, and engagement with local leaders can help build trust in the vaccine. Additionally, strategies for increasing accessibility, particularly in rural and underserved areas, need to be explored to ensure equitable vaccine distribution. By integrating these approaches into vaccination strategies, the aim is to enhance public health outcomes and optimize herd immunity within the broader population.

## Conclusion

Understanding HPV transmission and control dynamics requires identifying critical factors influencing infection and recovery. This study highlights the importance of vaccinating younger age groups, particularly those aged 9–14, as it significantly reduces cervical cancer incidence in Tanzania. Early vaccination not only provides long-term benefits by easing future efforts for older populations but also underscores the need for stratified data collection to evaluate vaccination strategies and improve intervention planning. Recovery plays a pivotal role in reducing HPV prevalence and preventing cervical cancer progression, with vaccination supporting both prevention and recovery. However, gaps in understanding long-term outcomes and mortality rates emphasize the need for enhanced data through collaboration with national cancer registries and improved surveillance systems.

Strengthened monitoring of HPV infection rates, vaccination coverage, and recovery patterns, alongside incorporating cervical cancer registry data, can offer deeper insights into disease progression and refine intervention strategies. Vaccinating girls aged 9–14 remains central to HPV control, while adaptive strategies for older populations address residual risks. Expanding early detection, treatment, and follow-up care complements vaccination efforts, reducing the cervical cancer burden and curbing HPV transmission. Achieving herd immunity is critical for halting transmission and lowering cancer incidence, with increasing immunity ultimately reducing the intensity of future vaccination efforts and advancing HPV eradication.

The findings recommend expanding vaccination programs to include females over 14 years, as outlined in WHO guidelines, to enhance coverage and prevent infections in older age groups. Integrating vaccination with cervical cancer screening programs could maximize the impact of both interventions. Improved data collection, through partnerships with institutions like the Ocean Road Cancer Institute (ORCI) and the National Institute for Medical Research (NIMR) in Dar es Salaam, can address gaps, particularly in understanding cancer progression and mortality rates.

While we acknowledge inherent data uncertainties, such as insufficient coverage and inconsistent records, we recognize the potential biases these gaps may introduce. Although the model focuses on female vaccination, incorporating males into HPV transmission dynamics would provide a more comprehensive understanding. Future studies should adopt a gender-inclusive approach. Assumptions such as 100% vaccine efficacy, lifelong immunity, and homogeneous mixing were made for simplicity, but they may oversimplify real-world conditions. Addressing factors like varying vaccine efficacy, waning immunity, and breakthrough infections will enhance model accuracy. Additionally, addressing logistical challenges such as rural vaccine distribution and conducting cost-effectiveness analyses alongside cervical screening programs are crucial for developing robust strategies.

To capture long-term effects more effectively, fractional calculus, specifically Atangana, Baleanu, and Caputo fractional derivatives, can provide advanced modeling techniques. These derivatives offer a realistic representation of HPV transmission dynamics, capturing delayed impacts of vaccination over time. They also enhance understanding of cervical cancer progression, given that persistent HPV infection is its primary cause. As demonstrated by [61], [62], [63], [64], [65], fractional derivative improves predictions for long-term disease control and vaccination effectiveness, guiding better intervention strategies. Future studies can explore these frameworks to enhance modeling approaches and predictive accuracy.

## CRediT authorship contribution statement

**Sylas Oswald:** Conceptualization, Methodology, Software, Formal analysis, Visualization, Investigation and Writing – original draft. **Eunice Mureithi:** Conceptualization, Methodology, Formal analysis, Investigation, Writing – review & editing, Resources, Supervision. **Berge Tsanou:** Conceptualization, Methodology, Formal analysis, Investigation, Writing – review & editing, Resources, Supervision. **Michael Chapwanya:** Conceptualization, Methodology, Formal analysis, Investigation, Writing – review & editing, Resources, Supervision. **Kijakazi Mashoto:** Biology aspect visualization, Investigation, Manuscript editing. **Crispin Kahesa:** Biology aspect visualization, Investigation, Manuscript editing.

## Funding statement

This study was fully funded by Bill & Melinda Gates Foundation.

## Declaration of competing interest

The authors declare that they have no known competing financial interests or personal relationships that could have appeared to influence the work reported in this paper.

## Data Availability

The data supporting the findings of this study were sourced from published articles as in TableTable 1, while some parameters were estimated using synthetic data, all of which are properly documented within the manuscript.
